# New geochemical data for defining origin and distribution of mercury in groundwater of a coastal area in southern Tuscany (Italy)

**DOI:** 10.1007/s11356-023-25897-7

**Published:** 2023-02-18

**Authors:** Giuseppe Protano, Stefano Bianchi, Matteo De Santis, Luigi Antonello Di Lella, Francesco Nannoni, Massimo Salleolini

**Affiliations:** 1 Department of Physical, Earth and Environmental Sciences, University of Siena, Via del Laterino 8, 53100 Siena, Italy; 2 Follonica, Italy

**Keywords:** Mercury, Groundwater, Coastal areas, Saline water intrusion, Geochemical anomalies, Continental sediments

## Abstract

A geochemical study was conducted in a coastal plain in the Orbetello Lagoon area in southern Tuscany (Italy), acquiring new data on groundwater, lagoon water, and stream sediment for insights into the origin, distribution, and behaviour of mercury in a Hg-enriched carbonate aquifer. The main hydrochemical features of the groundwater are ruled by the mixing of Ca–SO_4_ and Ca–Cl continental fresh waters of the carbonate aquifer and Na–Cl saline waters of the Tyrrhenian Sea and Lagoon of Orbetello. Groundwater had highly variable Hg concentrations (< 0.1–11 μg/L) that were not correlated with the percentage of saline water, depth in the aquifer, or distance from the lagoon. This excluded the possibility that saline water could be the direct source of Hg in groundwater and responsible for release of the element through interaction with the carbonate lithologies of the aquifer. The origin of Hg in groundwater could be ascribed to the Quaternary continental sediments overlying the carbonate aquifer because i) high Hg concentrations were found in the continental sediments of the coastal plain and in the contiguous lagoon sediments; ii) waters from the upper part of aquifer had the highest Hg concentrations; iii) Hg levels in groundwater increased with increasing thickness of the continental deposits. The high Hg content in the continental and lagoon sediments is geogenic due to regional and local Hg anomalies and to sedimentary and pedogenetic processes. It can be assumed that i) water circulating in these sediments dissolves the solid Hg-bearing constituents and mobilises this element mainly as chloride complexes; ii) Hg-enriched water moves from the upper part of the carbonate aquifer due to the cone of depression generated by intense pumping of groundwater by fish farms in the study area.

**Supplementary Information:**

The online version contains supplementary material available at 10.1007/s11356-023-25897-7.

## Introduction

Natural sources and human activities can contribute to the abundance of mercury (Hg) in coastal areas, where this toxic element is mainly transported by rivers and accumulates in oceanic and marine sediments, water, and biota (Covelli et al. 2001; Saniewska et al. 2014; Fostier et al. 2016; Liu et al. 2021). In these environmental settings, groundwater can also undergo mercury enrichment due to alteration and dissolution of solid Hg-bearing constituents of aquifers (e.g. cinnabar, Fe oxyhydroxides, and organic matter) or human inputs mostly related to mining and industry (Barringer et al. 2006; Sun et al. 2015; Spyropoulou et al. 2022).

In a coastal area of southern Tuscany (Italy) that includes the Lagoon of Orbetello, high concentrations of Hg have been detected in groundwater used in fish farming and agriculture as well as for domestic and drinking purposes. This environmental issue has been the subject of various geochemical studies that have proposed different sources and processes regulating the concentrations, distribution, and behaviour of Hg in the groundwater of the Orbetello Lagoon area (Grassi and Netti 2000; Protano et al. 2000; Salleolini et al. 2005; Bianchi 2018; Pasquetti 2019; Pasquetti et al. 2020; De Santis 2021). According to Grassi and Netti (2000) the interaction of saline water with the solid Hg-bearing constituents of rocks and sediments hosting aquifers affected by seawater intrusion is the main process responsible for Hg release and mobilisation in the groundwater of some coastal aquifers of southern Tuscany, including the carbonate aquifer system in the Orbetello Lagoon area. Protano et al. (2000) assumed that the high Hg concentrations in groundwater of the Orbetello Lagoon area were due to upwelling of gaseous elemental mercury (Hg^0^) into the upper part of the carbonate aquifer system, enhanced by the cone of depression caused by intense pumping of water from numerous wells in this stretch of coast in southern Tuscany. More recently, Pasquetti et al. (2020) determined high Hg concentrations in the Quaternary continental deposits in a coastal plain of the Orbetello Lagoon area (Ansedonia coastal plain) and suggested that these recent deposits could be the source of the Hg in local groundwater. Mercury contamination by human activities, natural rise of Hg-rich geothermal fluids, and diffuse degassing of Hg from deep sources were excluded in this area (Protano et al. 2000; Bianchi 2018; Pasquetti et al. 2020).

In this view, a hydrogeochemical study was conducted on groundwater of the Ansedonia coastal plain in the Orbetello Lagoon area. The study was undertaken to obtain insights into the origin, distribution, and behaviour of Hg in this coastal sector of southern Tuscany. The specific objectives of the study were i) to identify the processes controlling the main hydrochemical features (e.g. physico-chemical properties and concentrations of major ions) of Hg-enriched groundwater; ii) to define in groundwater the relationships between levels/distribution of Hg and the main hydrochemical features, fresh and saline water mixing, depth in the aquifer, and distance from the Lagoon of Orbetello; iii) to assess a potential source of Hg in the study area; iv) to suggest possible mechanisms responsible for the release and mobilisation of Hg in local groundwater.

To pursue these objectives, new geochemical data on groundwater and stream sediments of the Ansedonia coastal plain and the water of the Lagoon of Orbetello were acquired. Physico-chemical properties (temperature, pH, electrical conductivity, redox potential), concentrations of major ions (Ca, Mg, Na, K, Cl, SO_4_, HCO_3_), and trace elements including mercury (Al, As, Hg, Fe, Mn, Sb) were measured in 177 groundwater samples collected in the period June 2017–March 2021 from 15 wells of a fish farm in the Ansedonia coastal plain. The same physico-chemical properties, major ions, and trace elements were also determined in lagoon water samples (*n* = 8) collected in the eastern sector of the lagoon near the Ansedonia coastal plain. Concentrations of As, Hg, and Sb were measured in stream sediment samples (*n* = 6) from watercourses in the study area.

## Study area

**Fig. 1:**
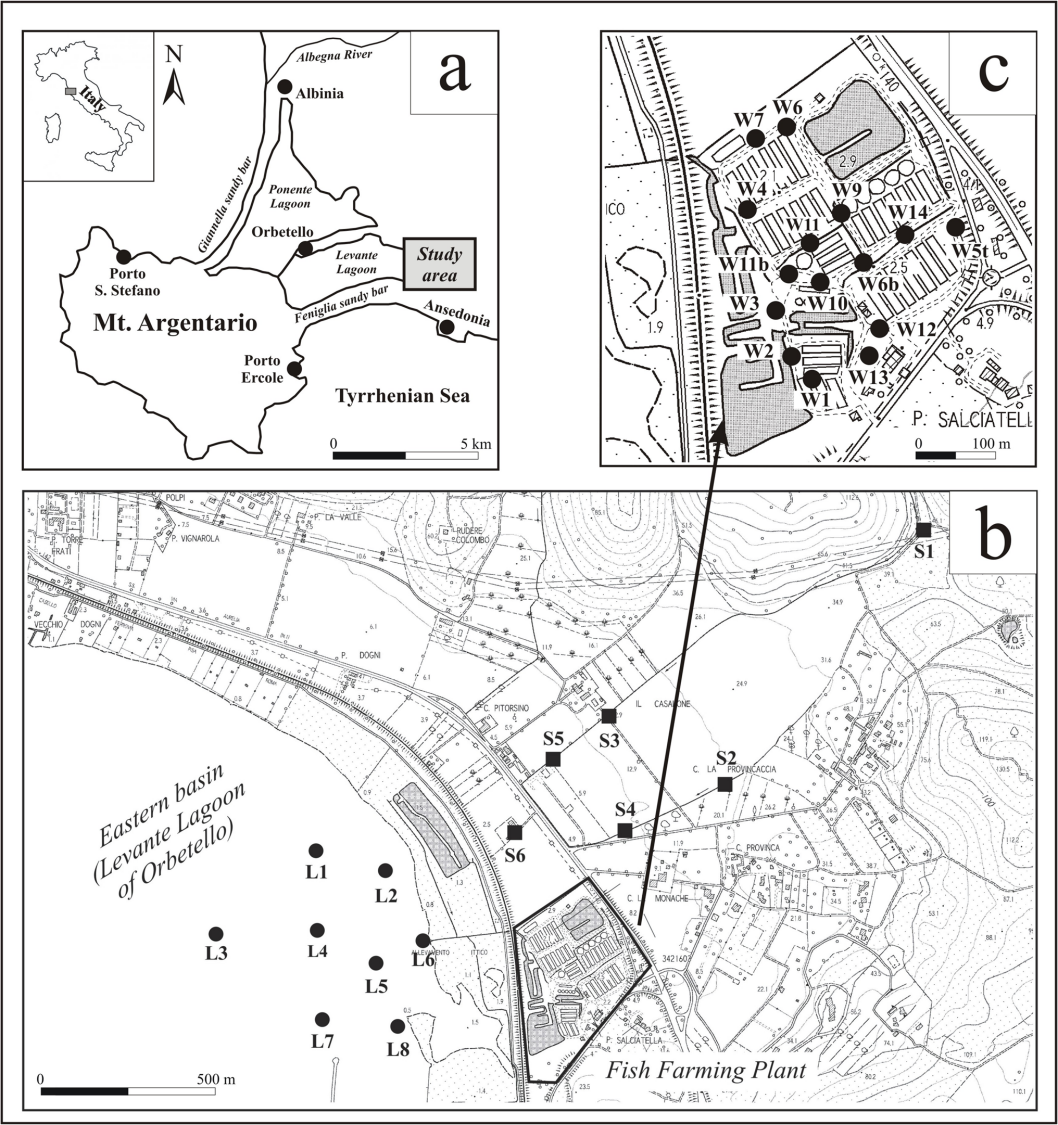
**a** The Orbetello Lagoon area along the Tyrrhenian coast of southern Tuscany (Italy) with location of the study area including the Ansedonia coastal plain (grey rectangle); **b** the Ansedonia coastal plain with location of the sampling sites of lagoon water (●) and stream sediment (■); **c** location of groundwater sampling wells in a fish farm in the Ansedonia coastal plain (●)

The study area is a coastal plain adjacent the eastern sector of the Lagoon of Orbetello on the Tyrrhenian coast of southern Tuscany, Italy (Fig. 1a). This coastal plain, hereafter named Ansedonia coastal plain, consists of Quaternary continental sediments that extend between hilly reliefs of carbonate rocks to the east and the lagoon to the west.

The Lagoon of Orbetello is a coastal lagoon about 27 km^2^ in area, enclosed by the Giannella sandy bar to the northwest, Mt Argentario promontory to the southwest, the Feniglia sandy bar to the south, and the coast of Tuscany to the northeast (Fig. 1a). Inside the lagoon there is a sandy strip about 4 km long and 400 to 600 m wide, where the town of Orbetello is located. The strip divides the lagoon into two hydrologically connected basins: the western basin (Ponente Lagoon) to the northwest with a surface of about 15 km^2^ and the eastern basin (Levante Lagoon) extending to the southeast for about 12 km^2^. The lagoon has an average depth of about 1 m and no sandbanks or tidal planes.

The Lagoon of Orbetello has little natural water exchanges with the Tyrrhenian Sea that occur through three artificial canals and are governed mainly by tides and winds. To increase water exchange in summer, artificial circulation is created by dewatering pumps which cause seawater to enter.

Depending on water exchange between lagoon and sea, evapotranspiration, fresh water entering from the Albegna River, direct precipitation, and surface runoff, the salinity of the lagoon water varies from 27.7‰ in winter to 47.4‰ in summer.

According to the classification of Thornthwaite (1948), the climate of the study area is subarid C_1_ (moisture index − 33.3 to 0). Annual average precipitation is about 700 mm, temperature 15.5 °C, and effective precipitation about 200 mm/year (Barazzuoli et al. 1993).

Italian law classifies the Lagoon of Orbetello as a national reclamation site due to contamination of the lagoon environment (mainly sediments) by heavy metals and organic compounds from past mining and industrial activities. The mining site is on the Monte Argentario promontory near the western part of the eastern basin, while the industrial one is on the coast near the eastern part of the western basin. In the mining site (Ferromin mine), a Fe–Mn mineralisation mainly consisting of limonite, hematite, and manganese oxides was exploited in the period 1872–1958 for producing manganese and iron oxides intended for the steel and metallurgical industries. In the industrial site the main plant (Sitoco) worked from 1908 to 1985 for manufacturing phosphate fertilisers.

Four fish farms operate around the lagoon, three of which in the Ansedonia area.

### Geological features

The geological features of the Orbetello Lagoon area are mainly related to the formation and evolution of the northern tract of Apennine chain. In southern Tuscany the Apennine orogenesis determined the translation and overlapping of tectonic units from different paleogeographic and sedimentation basins. From bottom to top, these tectonic units are the metamorphic Tuscan Units (basement), non-metamorphic Tuscan Units (Tuscan Nappe), Sub-Ligurian Units, and Ligurian Units (Decandia et al. 2001; Carmignani et al. 2013). Since Lower-Middle Miocene, post-collisional extensional tectonics formed NW–SE graben-type basins where lacustrine to marine sediments of the Neogene-Quaternary succession (Tuscan Neoautochthonous) were deposited (Bossio et al. 1998). An intrusive and effusive magmatic activity characterised by an eastward space–time migration was associated with this distensive tectonic phase (Peccerillo et al. 2001). The magmatic events produced geothermal systems and triggered a widespread circulation of hydrothermal fluids responsible for the formation of sulphide mineralisations (Tanelli and Lattanzi 1983; Lattanzi 1999; Dini 2003) among which the Hg mineralisations of the Monte Amiata area (Rimondi et al. 2015).

About the study area, the geology of the Ansedonia coastal plain consists of the Calcare cavernoso formation (fm), belonging to the non-metamorphic Tuscan Units, and Quaternary continental deposits of the Neogene-Quaternary succession (Fig. S1 in Supplementary Information).

The Calcare cavernoso fm (Upper Triassic) is a tectonic and autoclastic breccia consisting of elements of limestone and dolomitic limestone cemented by calcite. This carbonate formation resulted by an intense and complex transformation of the Burano fm formed by a sequence of layers of dark-grey dolomite and white anidrite. The main processes that transformed the evaporitic sequence of the Burano fm in the carbonate lithologies of the Calcare cavernoso fm were anhydrite hydration, gypsum dissolution, dolomite transformation, and calcite precipitation. The Calcare cavernoso fm widely outcrop in the study area (CCA in Fig. S1) with a maximum thickness of about 600 m (Salleolini et al. 2005).

In the study area, the Quaternary continental deposits of the Neogene-Quaternary succession form a coastal plain enclosed between the outcroppings of the Calcare cavernoso fm to the east, and the eastern basin of the Lagoon of Orbetello to the west. These continental deposits cover the lithologies of the Calcare cavernoso fm with a maximum thickness of about 30 m. The oldest Quaternary continental deposits (Middle-Upper Pleistocene) are dunes consisting of stratified and partially cemented brown to red sands (SSD in Fig. S1). The Pleistocene dunes are overlayed by Holocene deposits mostly including i) aeolian coastal dunes (ACD) consisting of yellowish sands and silts forming the Feniglia sandy bar and dune cordons near the eastern basin of the lagoon; ii) eluvial deposits (ELD) represented by heterometric clasts in a clayey and/or sandy matrix, and sandy-silty residual soils resulting from the weathering of the limestone and dolomitic limestone of the Calcare cavernoso fm.

### Hydrogeological features

In the Ansedonia coastal plain, as well as in the Orbetello Lagoon area, the most important aquifer system is hosted by the carbonate lithologies of the Calcare cavernoso fm located east of the lagoon. This formation is characterised by a high permeability due to a very porous matrix (primary permeability) and an intense tectonic fracturing and karstification (secondary permeability; Bianchi et al. 2011; Nocchi and Salleolini 2013). The carbonate aquifer is above an impermeable substrate consisting of Triassic quartzites and phyllites of the metamorphic Tuscan Units and extends to a maximum depth of 350–400 m in the Ansedonia area. The top of aquifer corresponds to the topographic surface where the Calcare cavernoso fm crops out or the bottom of the Quaternary continental deposits.

The carbonate aquifer system is mainly recharged by the direct infiltration of rainwater and, to a lesser extent, by a contribution from the regional flow of groundwater (lateral flow) in the northeastern sector. The natural outflow of aquifer is from northeast to southwest towards the Tyrrhenian Sea and Lagoon of Orbetello. The flow along the fresh water/saltwater interface is complex due to the upwelling of waters of different ages, depths, salinities, and temperatures.

In the Ansedonia coastal plain, a multi-aquifer system is hosted by the Quaternary continental deposits overlying the Calcare cavernoso fm. The recharge occurs through direct infiltration of meteoric waters while the natural discharge towards the Tyrrhenian Sea and Lagoon of Orbetello.

As in other coastal sectors of Tuscany (Barazzuoli et al. 1999, 2008; Grassi et al. 2007; Franceschini and Signorini 2016), in the study area the aquifers are affected by salinisation due to intrusion of waters from the Tyrrhenian Sea and Lagoon of Orbetello (Salleolini et al. 2005; Bianchi et al. 2011; Nocchi and Salleolini 2013). The mixing between fresh and saline waters is enhanced by the intense pumping of groundwater from the carbonate aquifer (Fig. S2 in Supplementary Information) mainly used for fish farming (about 48⋅10^6^ m^3^/year in the Ansedonia area) and domestic purposes (about 7⋅10^6^ m^3^/year; Nocchi and Salleolini 2013). The overexploitation of the carbonate aquifer has led to a degradation of chemical quality of groundwater mainly consisting in an increase of concentrations of chloride, sulphate, and sodium ions.

### Mercury in groundwater

Since the end of the 1990s, high concentrations of Hg have been detected in groundwater of the Orbetello Lagoon area used in fish farming and agriculture and for domestic and drinking purposes. They often exceeded 1 µg/L, the contamination threshold for Hg in groundwater (Italian Legislative Decree 152/2006), the limit in drinking water (Italian Legislative Decree 31/2001), and the threshold for the good chemical status of groundwater (Italian Legislative Decree 30/2009).

Various studies have been carried out in the Orbetello Lagoon area to determine the origin of the Hg and the processes responsible for its enrichment and distribution in groundwater (Grassi and Netti 2000; Protano et al. 2000; Salleolini et al. 2005; Bianchi 2018; Pasquetti 2019; Pasquetti et al. 2020; De Santis 2021).

Grassi and Netti (2000) focussed on clastic and carbonate aquifers along the Tyrrhenian coast of southern Tuscany including the Orbetello Lagoon area. They found that groundwater with high Hg concentrations was affected by seawater intrusion and showed a positive correlation between Hg and chloride ion concentrations. These findings suggested that seawater intrusion was the main process responsible for Hg release, mobilisation, and enrichment in groundwater as a consequence of the interaction between saline water and solid Hg-bearing constituents of the rocks and sediments hosting some coastal aquifers in southern Tuscany. The study also showed that water salinity, redox conditions, and pH play a key role in the dissolution of solid constituents containing Hg (e.g. cinnabar) and solubilisation of this element mainly as stable aqueous species with the chloride ion (e.g. HgCl_3_^−^, HgCl_2_^−^, HgCl_4_^2−^).

The hydrogeochemical study by Protano et al. (2000) excluded the possibility that the high Hg concentrations in groundwater of the Orbetello Lagoon area were due to i) contamination caused by human activities; ii) natural upwelling of geothermal fluids and/or diffuse degassing from deep sources connected with geothermal and hydrothermal activity in southern Tuscany (Batini et al. 2003; Brogi et al. 2016); iii) Hg release from carbonate lithologies of the Calcare cavernoso fm due to leaching by saline water intruding from the Tyrrhenian Sea. Conversely, the study assumed that Tyrrhenian seawater was the source of Hg in groundwater of the Orbetello Lagoon area, as this water has anomalous Hg concentrations (0.2–0.3 μg/L as compared to the normal level of 0.03 μg/L in seawater; Haynes et al. 2015) and intrudes the aquifers of this coastal sector of Tuscany. However, according to Protano et al. (2000) the high Hg concentrations in groundwater of the Orbetello Lagoon area were not due to a simple intrusion of Hg-enriched seawater but were probably determined by the following processes: i) intrusion of Hg-enriched Tyrrhenian seawater, in which Hg mainly occurs as stable and soluble complexes with the chloride ion (e.g. HgCl_4_^2−^), into the carbonate aquifer; ii) reduction of Hg^2+^ of chloride complexes to gaseous elemental mercury (Hg^0^) in the deepest part of the carbonate aquifer; iii) upwelling of Hg^0^ into the upper part of carbonate aquifer, enhanced by the cone of depression caused by intense water withdrawal from many wells in the Orbetello Lagoon area. The gaseous mercury presumably rises, associated with ultrafine particles in suspension and microbubbles in groundwater.

Salleolini et al. (2005) carried out a hydrogeological and hydrogeochemical study in the Orbetello Lagoon area and showed that the highest Hg concentrations (> 1 μg/L) were in groundwater of the Ansedonia coastal plain.

The geophysical, hydrogeological, and hydrogeochemical survey performed by Bianchi (2018) in the Ansedonia coastal plain excluded a source of Hg due to upwelling of deep waters and geothermal fluids along faults. Geophysical data showed that metals, possibly including Hg, were preferentially concentrated in the Quaternary continental deposits, suggesting that these sediments could be the source of Hg in local groundwater. In confirmation of this, high Hg contents up to 14.9 mg/kg were determined in continental sediment samples collected down to a depth of 6 m. Bianchi (2018) therefore concluded that the high Hg concentrations measured in groundwater from superficial wells of a fish farm in the Ansedonia coastal plain may be attributed to the capacity of these wells to drain Hg-rich water in the Quaternary continental deposits.

Pasquetti (2019) reported that in the Ansedonia coastal plain, the highest Hg levels were recorded in groundwater from superficial wells, suggesting that enrichment of Hg in the upper part of the carbonate aquifer could be due to the contribution of water circulating in the recent continental sediments that overlay the Calcare cavernoso fm.

Lastly, the sedimentological, mineralogical, and geochemical study of Pasquetti et al. (2020) determined the concentrations and mobility of Hg (and As) in the Quaternary continental sediments of the Ansedonia area in order to identify any Hg geochemical anomaly. Indeed, high Hg concentrations were found in the Late Pleistocene continental deposits of Ansedonia coastal plain and were considered a potential source of Hg in the local carbonate aquifer.

## Materials and methods

### Sampling

Groundwater samples (*n* = 177) were collected from wells of a fish farm located in the Ansedonia coastal plain close to the eastern part of the eastern basin of the Lagoon of Orbetello (Fig. 1c). In detail, 165 groundwater samples were taken by the CAIM Analysis Laboratory (Follonica, Italy) from 15 wells (wells W1 to W14) of the fish farm. The sampling was carried out every 3 or 4 months in the period June 2017–January 2021 as part of a monitoring program of waters used for fish farming. Two types of water sample were taken: 1) unfiltered and unacidified (NF-NA) water samples for the analysis of major anions; 2) filtered and HNO_3_-acidified (F-HNO_3_/A) water samples for the analysis of major cations and trace elements. The F-HNO_3_/A water samples were microfiltered at 0.45 µm and acidified by ultrapure HNO_3_ up to pH < 2.

In addition, 12 groundwater samples were taken by the Department of Physical, Earth and Environmental Sciences, University of Siena (Italy) from 7 wells of the fish farm in October 2020 and March 2021. The NF-NA and F-HNO_3_/A water samples were collected as above illustrated together with filtered and HCl-acidified water samples for analysis of mercury (F-HCl/A). The F-HCl/A water samples were microfiltered at 0.45 µm and acidified adding 1% (v:v) of ultrapure HCl (US EPA 1631-E/2002 method).

The groundwater sampling activity was complemented with in-field measurements of physico-chemical properties such as temperature, pH, electrical conductivity, and redox potential.

Lagoon water samples (*n* = 8) were collected in the eastern part of the eastern basin of the Lagoon of Orbetello (Fig. 1b). The sampling was carried out by the Department of Physical, Earth and Environmental Sciences, University of Siena (Italy), in November 2020. Unfiltered and unacidified (NF-NA), filtered and HNO_3_-acidified (F-HNO_3_/A), and filtered and HCl-acidified (F-Cl/A) water samples were collected at a depth of about 20 cm. Temperature, pH, and electrical conductivity of lagoon waters were measured in field.

Stream sediment samples (*n* = 6) were collected along two watercourses flowing in the Ansedonia coastal plain down to the eastern basin of the lagoon (Fig. 1b). The sampling of stream sediments was carried out by the Department of Physical, Earth and Environmental Sciences, University of Siena (Italy), in February 2020, collecting the first 10 cm of this geomaterial after removing any oxidation coating and plant covering. In each sampling site, a composite sample was taken by sampling equal quantities of stream sediment in 10 sub-sites located along 10 m of watercourse.

### Sample treatment and analysis

In the laboratory, groundwater and lagoon water samples were stored in the refrigerator at + 4 °C until the chemical analysis.

The stream sediment samples were dried at room temperature and sieved using a 150-µm sieve. The particle size fraction less than 150 µm was homogenised by manual quartering and mechanical pulverisation. About 250 mg of the pulverised stream sediment sample was solubilised by acid digestion adding the following ultrapure reagents: 2 mL HCl, 2 mL HNO_3_, 1 mL HF, and 1 mL HClO_4_ (US EPA 3052:1996 method). Solubilisation was performed in Teflon bombs using a Milestone ETHOS 900 microwave lab station.

Concentrations of major ions (Ca, Mg, Na, K) and trace elements (Al, As, Hg, Fe, Mn, Sb) in groundwater and lagoon water samples were measured by inductively coupled plasma–mass spectrometry (ICP-MS) according to the ISO 17294–2:2016 method. As for the major anions, chloride and sulphate concentrations were determined by liquid phase ion chromatography (ISO 10304–1:2009 method), while carbonate/bicarbonate concentrations by means of the acid–base colorimetric titration technique applying the APAT CNR IRSA 2010B method (manual 29/2003).

Concentrations of As, Hg, and Sb in stream sediment samples were determined by inductive coupled plasma–mass spectrometry (ICP-MS) according to the US EPA 6020B/2014 method.

### Data analysis

Analytical data of groundwater samples were preliminarily validated identifying and eliminating extreme outliers. The values of the physico-chemical properties as well as major ion and trace element concentrations higher than [75th percentile + (3 × IQR)] or lower than [25th percentile − (3 × IQR)], where IQR is the interquartile range given by the difference between the 75th and 25th percentiles, were considered extreme outliers.

The validated analytical data of groundwater samples were treated by univariate statistical analysis for calculating the main statistical indices such as minimum, maximum, mean, standard deviation, and 25th, 50th (median), and 75th percentiles. Concentrations of analytes below the respective instrumental detection limit were entered as a value equal to half this limit.

The correlations between the physico-chemical properties, major ion, and trace element concentrations in groundwater were defined by means of principal component analysis (PCA) using the XLStat software.

The Aquachem 3.7 software was used to realise Piper diagrams in order to establish the hydrochemical facies of groundwater and to calculate the mixing ratio between fresh and saline waters in groundwater.

The Surfer 11 software was utilised to create distribution maps of Hg concentrations in groundwater and the variation pattern of thickness of Quaternary continental sediments in the sector of the Ansedonia coastal plain where the fishing farm is located.

## Results and discussion

### Groundwater chemistry

#### Physico-chemical properties and major ions

As shown in Table 1, the physico-chemical properties and major ion concentrations of groundwater from the Ansedonia coastal plain varied widely despite the small sampling area (about 0.12 km^2^ inside the fish farm; Fig. 1c). With regard to the physico-chemical properties, pH and electrical conductivity were in the ranges 6.5–7.3 and 8440–62,111 µS/cm, respectively, indicating that the groundwater was prevalently very highly saline and neutral. The concentrations of major ions varied by an order of magnitude: Cl (2842–22,666 mg/L), Na (1313–11,636 mg/L), SO_4_ (657–6094 mg/L), Ca (289–1774 mg/L), Mg (186–1259 mg/L), and K (39–559 mg/L), with the exception of HCO_3_ (171–336 mg/L). The order of abundance was Na > Ca > Mg > K for cations and Cl > SO_4_ > HCO_3_ for anions.

**Table 1:** Statistical indices of physico-chemical properties and major ion concentrations in groundwater of the study area

	T (°C)	pH	EC (µS/cm)	Eh (mV)	Na (mg/L)	K (mg/L)	Ca (mg/L)	Mg (mg/L)	Cl (mg/L)	HCO_3_ (mg/L)	SO_4_ (mg/L)
*N*	177	174	173	165	175	168	174	175	175	177	173
min	17.7	6.5	8440	52	1313	39	289	186	2842	171	657
max	27.2	7.3	62,111	253	11,636	559	1774	1259	22,666	336	6094
mean	22.0	6.9	26,896	147	5246	183	786	593	9881	268	1915
SD	1.6	0.2	11,858	43	2471	99	281	253	4553	33	875
25^th^ percentile	20.9	6.9	17,720	112	3272	116	588	395	6122	250	1314
50^th^ percentile	21.7	7.0	24,500	154	4816	162	690	534	9120	275	1769
75^th^ percentile	22.6	7.1	32,400	176	6654	231	922	761	12,211	288	2150

*N*, number of data; *EC*, electrical conductivity at 20 °C; *Eh*, redox potential; *SD*, standard deviation

Despite the high variability of major ion concentrations, the groundwater belonged to a single sodium-chloride (Na–Cl) hydrochemical facies (Fig. S3 in Supplementary Information).

**Fig. 2:**
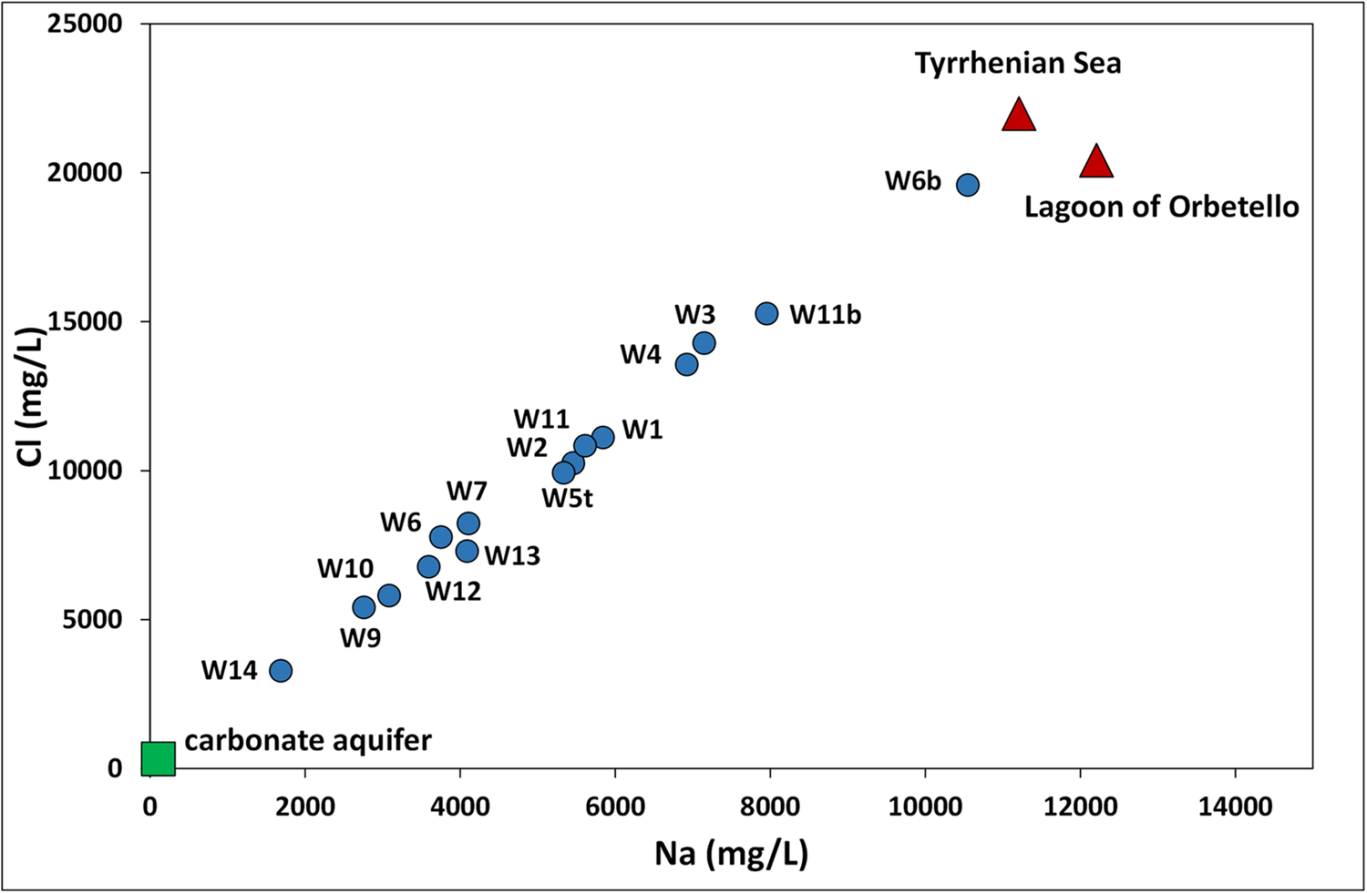
Correlation plot showing the distribution of Na and Cl concentrations in groundwater of the study area (dots), continental fresh water of the carbonate aquifer (square), and saline waters of the Tyrrhenian Sea and Lagoon of Orbetello (triangles) along a single mixing line

The high variability of electrical conductivity and major ion concentrations and the uniformity of the hydrochemical facies of groundwater can be explained by the variable mixing ratio between fresh and saline waters. The Na–Cl correlation plot of Fig. 2 showed that the groundwater of the Ansedonia coastal plain is arranged along a single mixing line and the end members of the mixing process are i) the calcium-sulphate (Ca–SO_4_) and calcium-chloride (Ca–Cl) continental fresh waters of the carbonate aquifer hosted by the Calcare cavernoso fm, showing the lowest Na and Cl concentrations (Salleolini et al. 2005); ii) the waters of the Tyrrhenian Sea and the Lagoon of Orbetello, showing the highest Na and Cl concentrations (Salleolini et al. 2005). It is known from previous studies that the coastal stretch of southern Tuscany including the Orbetello Lagoon area is subject to severe marine intrusion (Salleolini et al. 2005; Bianchi et al. 2011; Nocchi and Salleolini 2013).

Calculation of the mixing ratio between the above fresh and saline waters indicated that the percentage of saline water in the groundwater of Ansedonia coastal plain varies widely from 14 (well W14) to 90% (well W6b) and is mostly less than 50%.

**Fig. 3:**
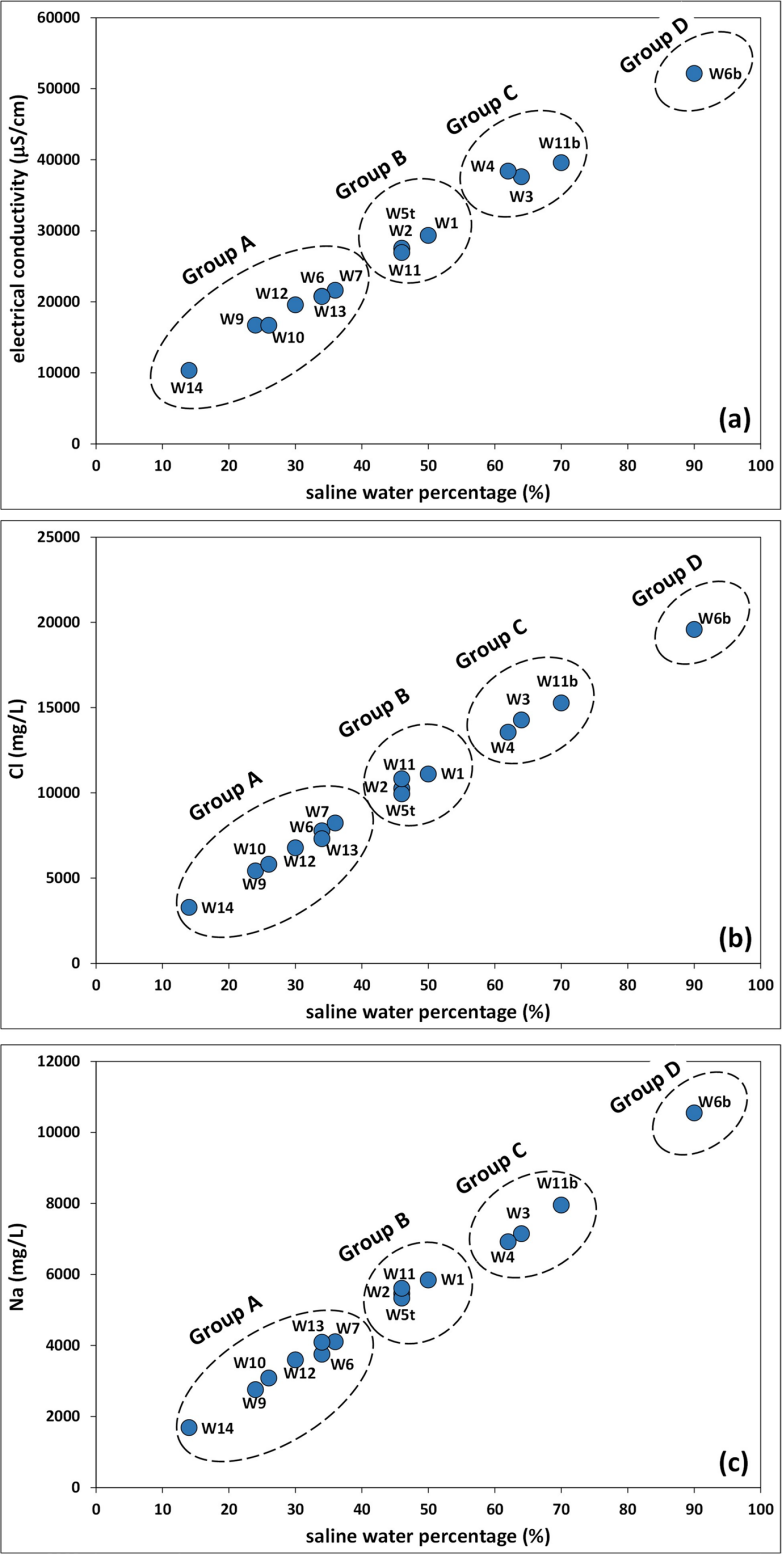
Correlation plots between the saline water percentage and electrical conductivity (**a**), Cl concentrations (**b**), and Na concentrations (**c**) in groundwater of the study area

Based on the percentage of saline water and concentrations of major ions, the groundwater of the Ansedonia coastal plain can be classified in four hydrochemical groups named groups A, B, C, and D (Fig. 3; Table S1 in Supplementary Information).

The water of group A was from superficial wells W6, W7, W9, W10, W12, W13, and W14 (depth 28 to 44 m) in the central and eastern sectors of the sampling area. This water had a percentage of saline water between 14 and 36% and the lowest electrical conductivity values and major ion concentrations. Group B water was from superficial wells W1, W2, and W11 (depth 28 to 34 m) situated in the central and southwestern sectors of the sampling area, as well as from the deep well W5t (129 m). The percentage of saline water in group B samples was in the narrow range 46–50%. Group C water was from superficial wells W3 and W4 (depths 18 and 40 m, respectively) in the western sector of the sampling area, and from the deep well 11b (110 m). It contained a percentage of saline water in the range 62 to 70%. Group D water was from the deep well W6b (153 m) and showed the highest percentage of saline water (90%), electrical conductivity values, and major ion concentrations.

**Fig. 4:**
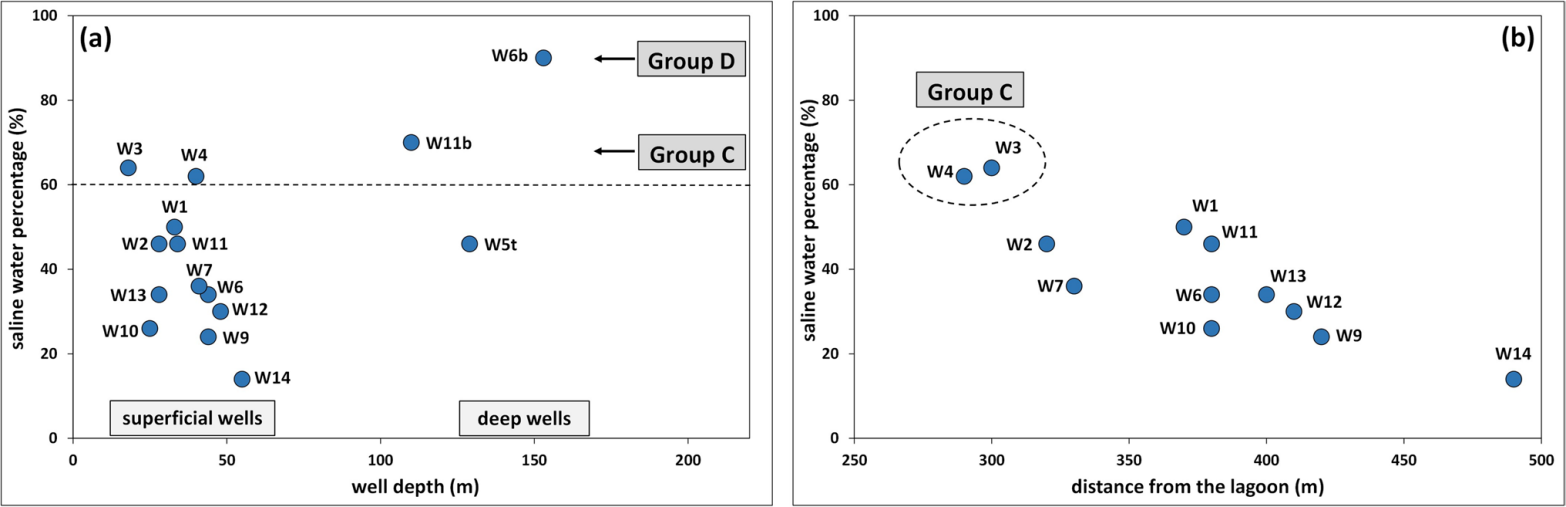
Correlation plots between the saline water percentage in groundwater and depth of sampling wells (**a**) and distance of surface wells from the eastern basin of the Lagoon of Orbetello (**b**)

**Fig. 5:**
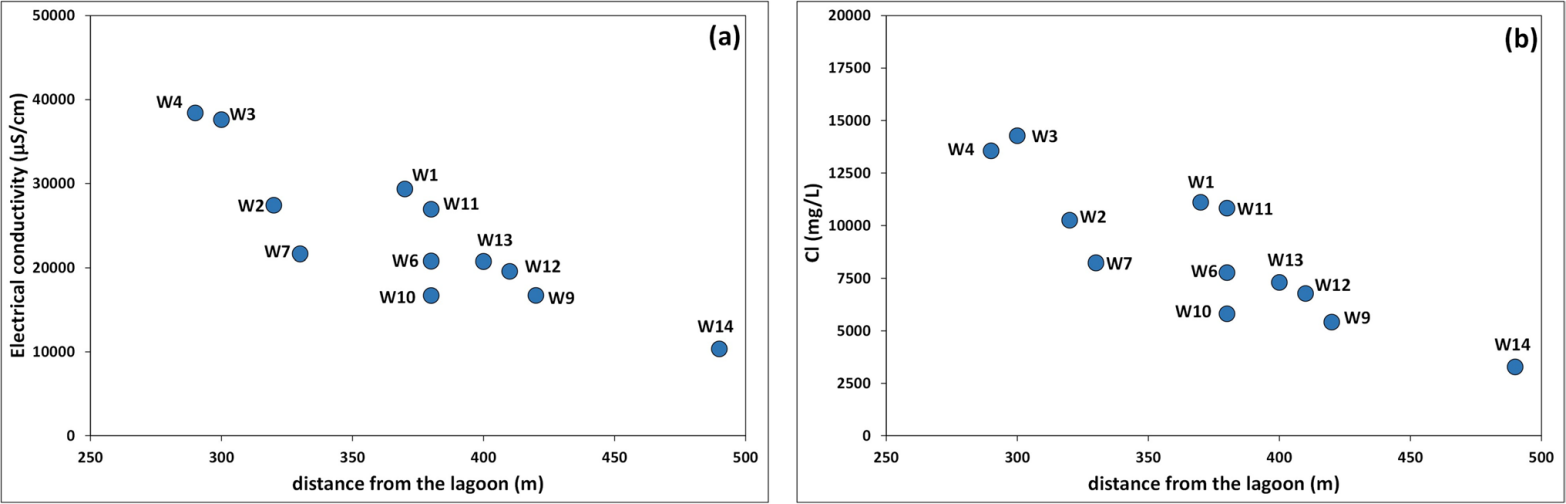
Variation of electrical conductivity (**a**) and Cl concentration (**b**) in groundwater from surface wells as a function of the distance from the eastern basin of the Lagoon of Orbetello

The hydrochemical features of groundwater belonging to groups A, B, C, and D highlighted that the intrusion of saline water in the Ansedonia coastal plain is ruled by depth in the carbonate aquifer and distance from the Lagoon of Orbetello. In fact, saline water intrusion was more severe in groundwater of groups D and C from the deepest wells (depth more than 100 m, except well W5t) and from superficial wells (depth 18 to 44 m) closest to the lagoon (Fig. 4). Reflecting the level of saline intrusion, electrical conductivity and concentrations of major ions in water from the superficial wells gradually decreased from west to east from the eastern basin of the lagoon towards the hinterland (Fig. 5). As a whole, these findings indicate that the chemistry of groundwater in the deep part of the carbonate aquifer is mainly influenced by intrusion of Tyrrhenian seawater, while in the superficial part, input of saline water from the lagoon is predominant, presumably facilitated by the intense pumping of water from wells by the fish farms in the Ansedonia area.

#### Trace elements

Among the trace elements analysed, Al, Fe, and Mn showed highly variable concentrations in groundwater sampled from the 15 wells of the fish farm in the period June 2017–March 2021: Al = 1.07–49.9 μg/L, Fe = 4–54.9 µg/L, Mn =  < 1–342 µg/L (Table 2). Within these wide intervals, Al, Fe, and Mn concentrations were mostly below 10, 15, and 10 μg/L, respectively. On the contrary, As and Sb concentrations in groundwater collected from 7 wells of the fish farm in October 2020 and March 2021 were more homogeneous: 0.38–0.98 and 0.23–0.81 µg/L, respectively (Table 2). They were comparable to those determined by Pasquetti (2019) in groundwater from 12 wells of the fish farm in June 2018: As = 0.5–1.4 µg/L, Sb = 0.1–0.8 µg/L. As a whole, this analytical data indicated that As and Sb concentrations in groundwater of the study area are within the normal range of their natural levels in groundwater (Filella et al. 2002; Smedley and Kinniburgh 2002). This finding suggests that in the Ansedonia coastal plain, there is no circulation of geothermal fluids enriched in As and Sb (as well as Hg), as occurs in other areas of Tuscany (Grassi et al. 2014).

**Table 2:** Statistical indices of Al, Fe, Mn, As, Sb, and Hg concentrations in groundwater of the study area

	Al (μg/L)	Fe (μg/L)	Mn (μg/L)	As (μg/L)	Sb (μg/L)	Hg (μg/L)
*N*	141	166	172	12	12	175
min	1.07	4.00	< 1	0.38	0.23	< 0.1
max	49.90	54.90	342	0.98	0.81	11.0
mean	5.35	13.17	11.43	0.63	0.43	2.48
SD	5.78	7.45	40.67	0.21	0.18	2.69
25th percentile	2.63	8.45	< 1	0.45	0.26	0.72
50th percentile	3.79	11.20	1.91	0.61	0.48	1.31
75th percentile	5.55	15.00	5.49	0.75	0.52	3.27

*N*, number of data; *SD*, standard deviation

As a rule, Al, Fe, and Mn concentrations in groundwater of the Ansedonia coastal plain increased as the percentage of saline water increased. On the other hand, the Al, Fe, and Mn levels in waters of the eastern part of the eastern basin of the lagoon (Fe = 15.6 μg/L, Mn = 27.05 μg/L on average; this study; Table S2 in Supplementary Information) and in the Tyrrhenian Sea (Al = 7.77 μg/L; Salleolini et al. 2005) were higher than those in the continental fresh waters of the carbonate aquifer (Al = 4.4 μg/L, Fe = 10.94 μg/L, Mn = 3.15 μg/L, on average; Salleolini et al. 2005). Conversely, analytical data from this study and from Salleolini et al. (2005) suggests that saline water of the Tyrrhenian Sea and Lagoon of Orbetello did not affect As and Sb concentrations in groundwater.

The Hg concentrations in groundwater of the Ansedonia coastal plain varied widely in the range < 0.1 to 11 μg/L (2.48 μg/L on average; Table 2). Water from wells W6 and W7 showed the highest Hg concentrations (10.3 and 8.16 μg/L on average, respectively), while that from wells W1, W5t, W6b, W12, and W13 showed the lowest concentrations (0.52 to 0.98 μg/L; Table 3). Mercury concentrations in groundwater frequently exceeded the contamination threshold (1 μg/L) established by Italian Legislative Decree 152/2006. This occurred in 109 out of 175 water samples mainly collected from wells W3, W4, W6, W7, W9, W10, W11, and W14.

**Table 3:** Statistical indices of Hg concentrations (in μg/L) in groundwater of the study area grouped by sampling well

	W1	W2	W3	W4	W5t	W6	W6b	W7
*N*	14	15	2	4	16	2	15	8
Min	< 0.1	0.25	2.08	1.09	< 0.1	10.10	< 0.1	2.56
Max	1.41	1.84	5.29	6.70	2.03	10.50	1.35	10.90
Mean	0.76	1.03	3.69	4.37	0.98	10.30	0.52	8.16
SD	0.37	0.44	–	2.39	0.56	–	0.41	2.78
	W9	W10	W11	W11b	W12	W13	W14	
*N*	15	14	15	16	16	14	9	
Min	0.52	0.58	< 0.1	0.17	< 0.1	< 0.1	1.26	
Max	11.00	4.78	9.02	2.84	1.94	1.67	7.54	
Mean	5.91	2.20	4.42	1.51	0.95	0.77	4.11	
SD	2.91	1.14	2.3	0.81	0.53	0.42	2.05	

*N*, number of data; *SD*, standard deviation

### Mercury origin, distribution, and behaviour in groundwater

**Fig. 6:**
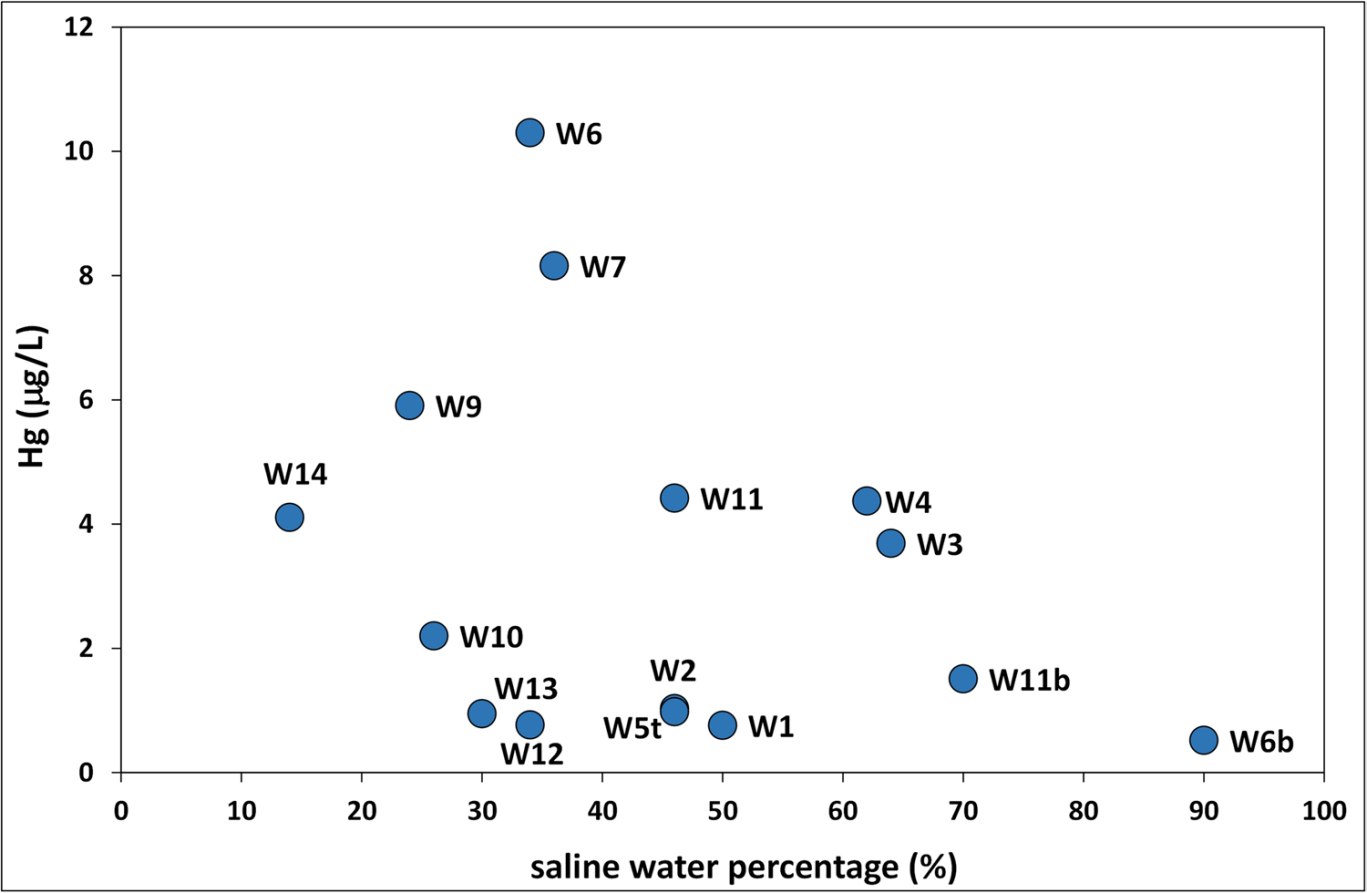
Correlation plot between the Hg concentrations and saline water percentage in groundwater of the study area

Unlike other coastal aquifers affected by seawater intrusion and Hg enrichment (Grassi and Netti 2000; Spyropoulou et al. 2022), no positive relationship was found between Hg levels and percentage of saline water or Cl and Na concentrations in groundwater of the Ansedonia coastal plain (Fig. 6). However, the analytical data indicated that average Hg concentrations differed as follows between hydrochemical groups: group A (3.4 μg/L) > group C (2.2 μg/L) > group B (1.8 μg/L) > group D (0.5 μg/L).

Regarding the influence of saline water on the abundance of Hg in groundwater of the study area, it should be noted that Hg concentrations in Tyrrhenian seawater near the Lagoon of Orbetello were as high as 0.3 μg/L (Protano et al. 2000; Salleolini et al. 2005). Although significantly above Hg concentrations in Mediterranean seawater from coastal sites and the open sea (< 0.02 μg/L; Fantozzi et al. 2013), Hg levels in local Tyrrhenian seawater were lower than those measured in most groundwater of the Ansedonia coastal plain. Analyses performed in this study also indicated that water of the eastern basin of the lagoon had low Hg concentrations (< 0.1 μg/L) except in the sector where a channel discharges wastewater from the fish farm into the lagoon (Hg = 0.15–0.49 μg/L; Table S2 in Supplementary Information).

On the whole, these findings excluded the possibility that saline waters from the Tyrrhenian Sea and Lagoon of Orbetello are the direct source of Hg in groundwater of the Ansedonia coastal plain, or that they may be involved in release of this element by interaction with the lithologies hosting the carbonate aquifer.

**Fig. 7:**
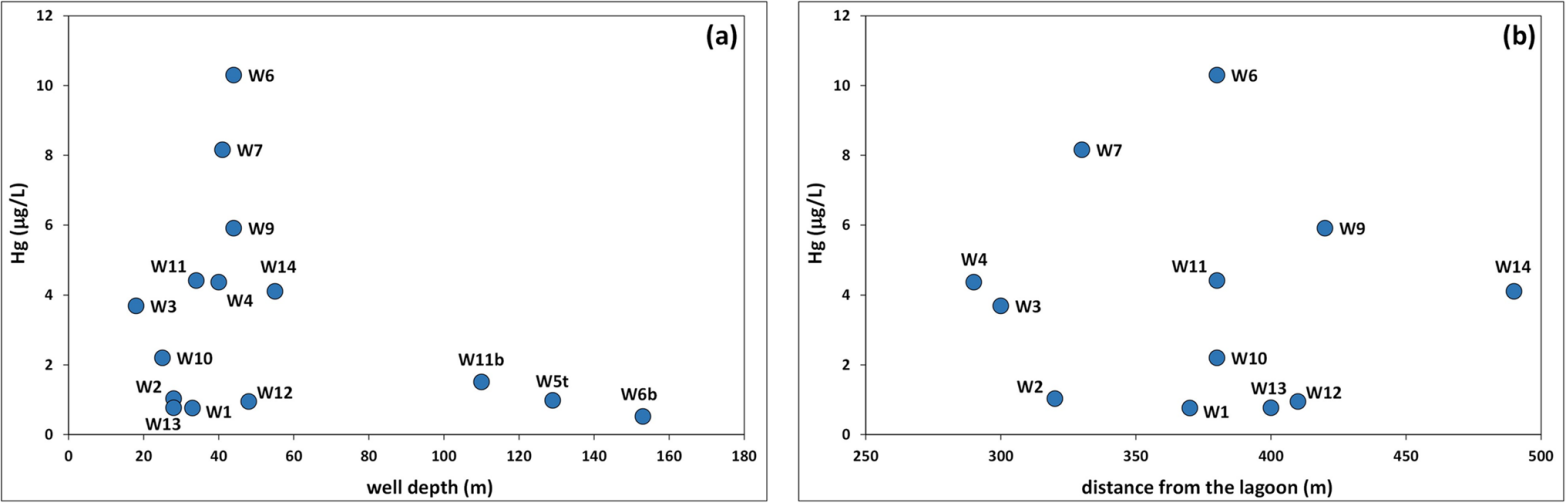
Correlation plots between the Hg concentrations in groundwater and depth of sampling wells (**a**) and distance of surface wells from the eastern basin of the Lagoon of Orbetello (**b**)

As confirmation of this, Hg concentrations in groundwater from the Ansedonia coastal plain were not correlated with well depth or well distance from the eastern basin of the lagoon (Fig. 7). In fact, Hg concentrations in water from the deep wells W11b, W5t, and W6b were rather homogeneous and decreased slightly from 1.51 to 0.52 μg/L (average values) with increasing well depth and saline water percentage. On the contrary, water from superficial wells showed highly variable Hg concentrations (average 0.76 to 10.3 μg/L) unrelated to saline water percentage, well depth, or distance from the lagoon (Fig. 7).

**Fig. 8:**
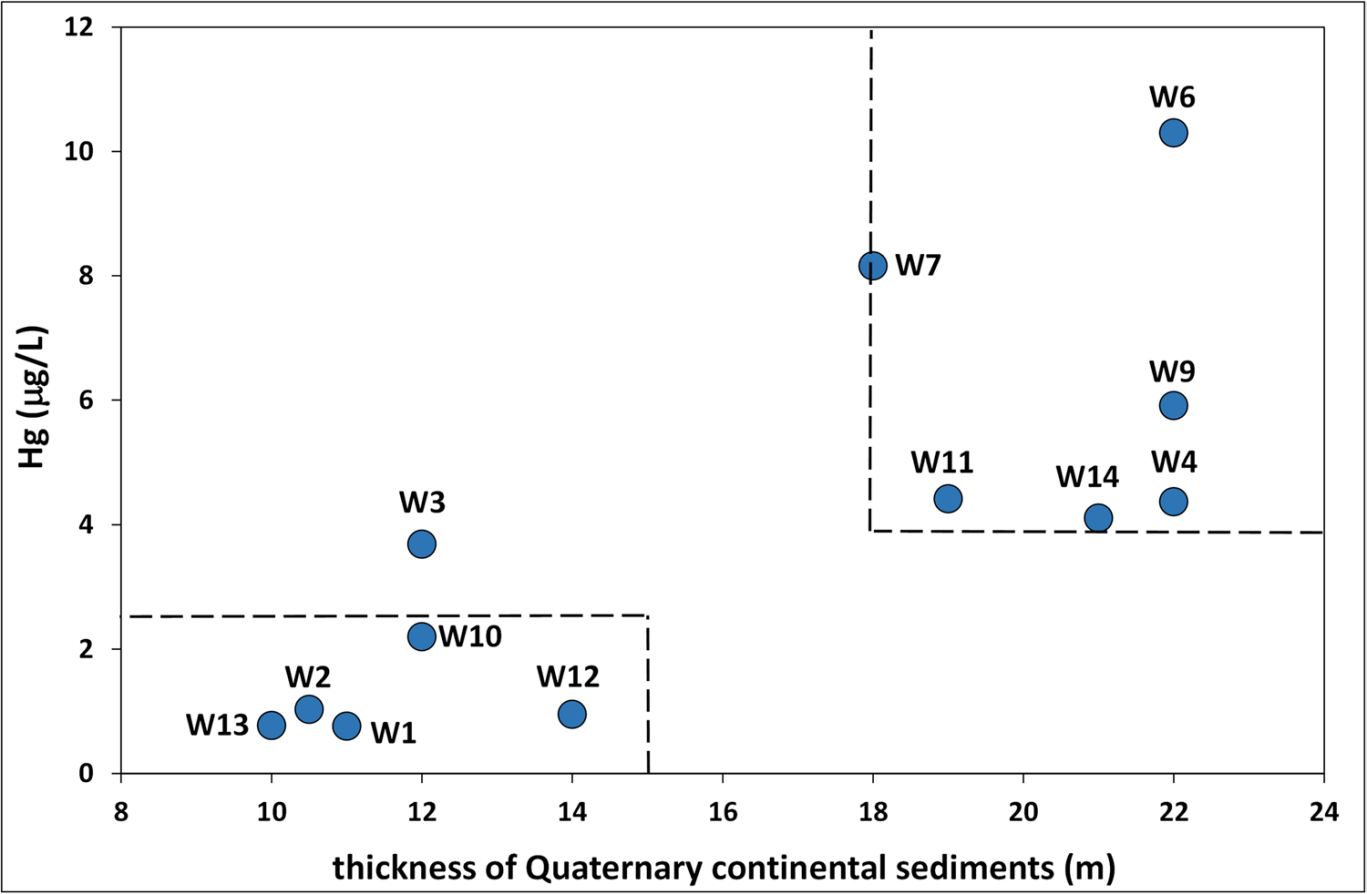
Relationship between the Hg concentrations in groundwater and thickness of the overlying Quaternary continental sediments

After excluding the possibility that Hg originated from Tyrrhenian seawater and lagoon water and their interactions with the lithologies of the carbonate aquifer system, the Quaternary continental deposits of the Ansedonia coastal plain were also investigated as the possible source of Hg in local groundwater. The available data indicated that Hg concentrations in groundwater from superficial wells increased with increasing thickness of the overlying continental sediments (the thickness of Quaternary continental sediments was established from stratigraphic data obtained from well logs acquired during drilling). As shown in Fig. 8, the highest Hg concentrations (> 4 μg/L on average) were measured in water from superficial wells perforating a thickness of Quaternary continental deposits from 18 to 22 m, whereas Hg levels were usually below 2.5 μg/L in water from superficial wells located where the cover of continental sediments was less thick (< 15 m).

**Fig. 9:**
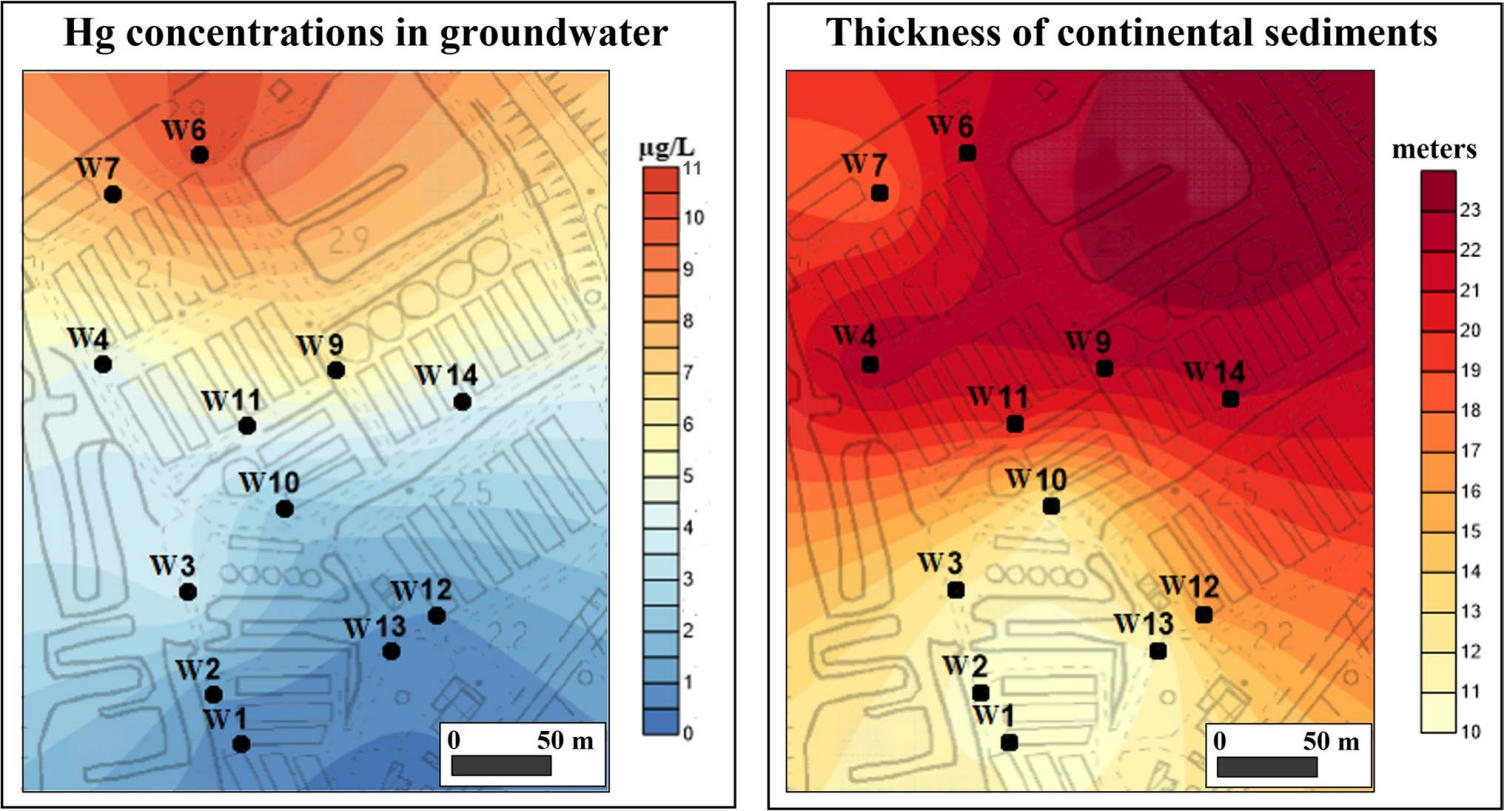
Maps showing the spatial distribution of the Hg concentrations in groundwater (average values) from the upper part of the carbonate aquifer, and the variation of thickness of Quaternary continental sediments in the study area

In line with the above, the spatial distribution of mercury in the upper part of the carbonate aquifer in the study area showed that Hg concentrations in groundwater decreased from north to south as did the thickness of the overlying Quaternary continental sediments (Fig. 9).

The results of this study agree with the findings of Pasquetti et al. (2020) who hypothesised that Hg enrichment in groundwater of the carbonate aquifer was due to Hg acquired by circulation of water in the Quaternary sediments of the Ansedonia coastal plain. This hypothesis arose from anomalous Hg concentrations measured in Late Pleistocene continental sediments and paleosols (0.2–2.7 mg/kg) that are significantly higher than Hg concentrations in the upper continental crust (0.056 mg/kg; Wedepohl 1995), magmatic and sedimentary rocks (0.01–0.4 mg/kg; De Vos and Tarvainen 2006), and uncontaminated soils (0.05 mg/kg; Reimann and de Caritat 1998). Moreover, Hg concentrations in these continental sediments exceeded the estimated geochemical background in southern Tuscany which is 0.2–0.3 mg/kg (Dall’Aglio et al. 1966; Baroni et al. 1994; Protano et al. 1998). Similar Hg concentrations in the continental deposits of the Ansedonia coastal plain (0.15–4.55 mg/kg, with a peak 14.9 mg/kg) were measured by Bianchi (2018); the author also reported detecting cinnabar crystals in these sediments.

**Fig. 10:**
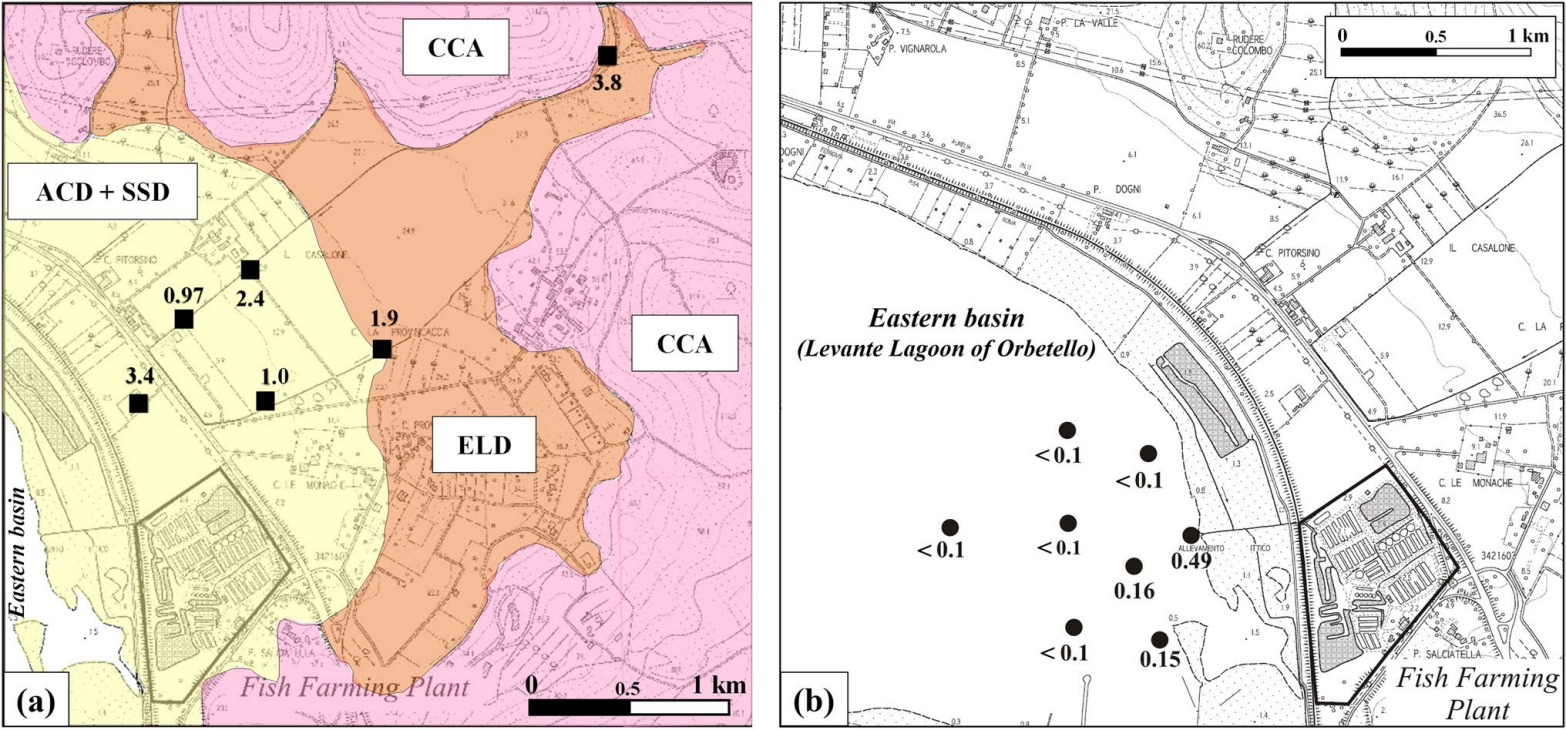
**a** Hg concentrations (in mg/kg) in stream sediments of the Ansedonia coastal plain (ELD, Holocene eluvial deposits and residual soils; ACD + SSD, Holocene aeolian coastal dunes and Middle-Upper Pleistocene stratified sandy dunes; CCA, Calcare cavernoso formation (Upper Triassic)); **b** Hg concentrations (in μg/L) in lagoon water of the eastern part of the eastern basin of the Lagoon of Orbetello

New geochemical data on stream sediments produced in the present study endorse the presence of a Hg anomaly in the study area. Indeed, Hg concentrations from 0.97 to 3.8 mg/kg were determined in stream sediment samples collected along two watercourses traversing the Ansedonia coastal plain and entering the eastern part of the eastern basin of the lagoon (Fig. 1b; Table S3 in Supplementary Information). As shown in Fig. 10a, these high Hg concentrations were measured in stream sediments mainly derived from Middle-Upper Pleistocene and Holocene dunes (ACD + SSD in figure), Holocene eluvial deposits, and residual soils (ELD) formed by the carbonate lithologies of the Calcare cavernoso fm. A geochemical survey by Rimin (1985) measured high Hg contents (1–5 mg/kg) in stream sediments of watercourses in large outcrops of the Calcare cavernoso fm to the east of the study area. In this regard, it should be noted that Fe–Mn oxide deposits and polymetallic sulphide mineralisation were found in the Calcare cavernoso fm on the nearby promontory of Monte Argentario (Burtet Fabris and Omenetto 1975).

Although the above findings show local mercury enrichment in the Calcare cavernoso fm, it can be excluded that this carbonate formation is the Hg source in groundwater of the Ansedonia coastal plain for the following reasons: i) no relationship was found in groundwater between Hg levels and major ion concentrations resulting from water–rock interactions in the carbonate aquifer, as shown by principal component analysis (Fig. S4 in Supplementary Information); ii) Hg variability in groundwater was unrelated to fluctuations of saline water intrusion; iii) low Hg concentrations (< 0.1 μg/L) were found in groundwater from wells that cross solely carbonate lithologies of the Calcare cavernoso fm in the hills surrounding the Ansedonia coastal plain (Salleolini et al. 2005).

The high Hg concentrations in the Quaternary continental sediments of the Ansedonia coastal plain can be considered natural due to i) regional and local geogenic sources of Hg related to geochemical anomalies of this element in southern Tuscany (Cossa and Coquery 2005); ii) sedimentary and pedogenetic processes (e.g. weathering, erosion, transport, sorting, and sedimentation) responsible for distribution and accumulation of Hg-rich particles in continental and marine sediments, as well as formation of Hg-enriched eluvial deposits and residual soils (Rimondi et al. 2012; Lattanzi et al. 2017).

The regional sources of Hg are mainly in the Monte Amiata area that is characterised by a diffuse Hg anomaly and world-class cinnabar (HgS) mineralisations (Ferrara et al. 1991; Rimondi et al. 2015). The Monte Amiata area is about 50 km northeast of the Lagoon of Orbetello and is drained by several streams such as the Albegna River that flow into the Tyrrhenian Sea near the Ansedonia coastal plain. The regional Hg sources in the Monte Amiata area determined the anomalous levels of this element in the Quaternary continental sediments in the pre-industrial period, that is before the mining and smelting of the cinnabar mineralisations of the Monte Amiata mercury district, which mainly happened from 1870 to 1974. The Monte Amiata cinnabar mineralisations underwent long and intense exploitation and ore processing that produced large quantities of waste accumulated in open-air dumps (Rimondi et al. 2015; Protano and Nannoni 2018). The Hg-enriched materials in the dumps were eroded, moved, and diffused into the environment by surface runoff and transport by watercourses (e.g. Albegna, Fiora and Paglia Rivers) flowing from the Monte Amiata area to the Tyrrhenian Sea (Gray et al. 2014; Lattanzi et al. 2017; Colica et al. 2019; Rimondi et al. 2019; Fornasaro et al. 2022). These processes involving waste in mining and smelting sites, cinnabar mineralised areas and mercury anomalous rocks, determined Hg enrichment in stream, overbank, and alluvial sediments (Dall’Aglio et al. 1966; Protano et al. 1998), as well as in marine sediments and waters of the sector of the Tyrrhenian coast embracing the Orbetello Lagoon area (Barghigiani et al. 1996; Leoni and Sartori 1996; Scanu et al. 2016).

The local source of Hg in the Quaternary continental deposits of the Ansedonia coastal plain could be the proximal outcroppings of the Calcare cavernoso fm as revealed by Hg-enriched eluvial deposits and residual soils derived from the carbonate lithologies of this lithostratigraphic unit.

The anomalous Hg levels in the Quaternary continental deposits of the study area can also explain the high concentrations of this element in the sediments of the eastern basin of the Lagoon of Orbetello (ISPRA 2009; Romano et al. 2015; Pasquetti 2019; Mancini et al. 2022). It should be noted that the highest Hg concentrations in the eastern basin (frequently > 2 mg/kg) were measured in sediments from the eastern part close the Ansedonia coastal plain and the western part near a former mining site (Ferromin mine). In these two zones, Hg concentrations normally reached the peak in the upper 20 cm of the lagoon sediment and were higher than the natural background in sediments of Lagoon of Orbetello estimated to be 0.87 mg/kg as upper natural concentration (Romano et al. 2015). The high concentrations of Hg found in sediments of the western part can be considered anthropogenic related to mining of the Fe–Mn oxide mineralisation and ore processing in the period 1958 to 1972. Conversely, natural sources and processes such as erosion, transport, and deposition of particles from the Quaternary continental sediments of the Ansedonia coastal plain are presumably responsible for the anomalous Hg concentrations in lagoon sediments from the eastern part of the eastern basin of the lagoon. Further, Hg in these lagoon sediments could come from wastewater from the fish farm in the Ansedonia coastal plain. Mercury concentrations in this wastewater are monitored and were below the threshold of 5 μg/L established by Italian Legislative Decree 152/2006 for wastewater discharged into superficial waters. As mentioned above, water samples collected in the eastern part of the eastern basin (Fig. 1b) showed the highest Hg concentrations (0.15–0.49 μg/L) close to the coastline near where the wastewater channel of the fish farm enters the lagoon (Fig. 10b; Table S2 in Supplementary Information). Moving away from the coastline, Hg concentrations in lagoon water were lower and constantly below 0.1 μg/L. Unlike Hg, waters of the eastern part of the eastern basin had rather homogeneous levels of Fe (10–22.7 μg/L), Mn (14.7–36.4 μg/L), As (0.11–1.49 μg/L), and Sb (0.61–1.03 μg/L).

Summing up, it can be assumed that i) the Hg-enriched Quaternary continental sediments of the Ansedonia coastal plain and lagoon sediments of the eastern part of the eastern basin are the main sources of Hg in groundwater of the study area; ii) the fresh and saline waters circulating in the continental and lagoon sediments could release mercury from the Hg-bearing minerals and organic compounds in these sediments, as well as mobilise and transport this element into the aquifer hosted by the underlying carbonate rocks; iii) the circulation of fresh and saline waters in the continental and lagoon sediments and their movement into the carbonate aquifer could be enhanced by the intense pumping of water (about 48⋅10^6^ m^3^/year) from the wells of the fish farms in the Ansedonia area (Fig. S5 in Supplementary Information).

It is therefore crucial to identify the factors and processes by which mercury is released from the Hg-bearing solid constituents of the continental and lagoon sediments and mobilised in the water circulating in these geomaterials. In this respect, Hg concentrations from 0.6 to 9.7 μg/L, which are less than 3% of the total element content, were measured by Pasquetti et al. (2020) in solutions from leaching tests on the Quaternary sediments of the Ansedonia coastal plain. While these values indicate that important amounts of Hg can be released by the continental sediments of the study area, they also suggest that in these sediments Hg is mainly associated with insoluble and less soluble minerals, such as cinnabar and Fe–Mn oxyhydroxides and organic compounds.

Various studies have suggested that dissolved organic matter (Ravichandran et al. 1998; Waples et al. 2005; Miller et al. 2009), oxidant conditions (Holley et al. 2007), microbial activity under oxic and anoxic conditions (Barkay and Wagner-Döbler 2005; Balland-Bolou-Bi et al. 2017), pH, and high-water concentrations of Fe^3+^ and sulphur chemical species, such as HS^−^ and S^0^ (Jay et al. 2000), are the major factors regulating the dissolution of cinnabar and metacinnabar (HgS). The dissolution of these Hg sulphides usually occurs by oxidation of sulphur (S^2−^), forming elemental Hg^0^ and Hg^2+^ water soluble species in relation to pH, redox conditions, and concentrations of ligands.

Mercury adsorbed on organic matter (e.g. humina and humic acids) and Fe–Mn oxyhydroxides can be released as a result of mineralisation of organic compounds (Cossa and Gobeil 2000) and weathering of oxyhydroxides by reduction of Fe^3+^ and Mn^4+^ (Harris-Hellal et al. 2011). Moreover, calcium ions (Ca^2+^) can exchange Hg^2+^ ions physically adsorbed on the surfaces of Fe oxyhydroxides and clay minerals (Gerbig et al. 2011).

The water solubility of Hg is mainly controlled by inorganic and organic ligands such as Cl^−^, OH^−^, and S^2−^ ions and dissolved organic matter (Gabriel and Williamson 2004). Among the inorganic ligands, the chloride ion has a key role in Hg mobilisation as it forms stable water-soluble complexes with Hg^2+^, such as HgCl_4_^2−^, HgCl_3_^−^, HgCl_2_, and HgClOH (Kim et al. 2004; Spyropoulou et al. 2022). Moreover, Hg can be adsorbed by dissolved organic matter (DOM) through complexation reactions involving the sulphur-, oxygen-, and nitrogen-bearing functional groups of DOM substances (Wallschlager et al. 1996; Ravichandran 2004; Liu et al. 2019).

## Conclusions

In a coastal plain of the Orbetello Lagoon area (Ansedonia coastal plain) in southern Tuscany (Italy), high concentrations of Hg have been detected in the groundwater of a carbonate aquifer mainly used by fish farms. The groundwater showed highly variable physico-chemical properties and concentrations of major ions, despite the fact that it belongs to a single sodium-chloride (Na–Cl) hydrochemical facies. Analytical data indicated that i) the main hydrochemical features of the groundwater are ruled by the mixing of calcium-sulphate (Ca–SO_4_) and calcium-chloride (Ca–Cl) continental fresh waters of the carbonate aquifer and saline waters of the Tyrrhenian Sea and Lagoon of Orbetello; ii) the mixing ratio of fresh saline waters depends mainly on depth in the aquifer and distance from the lagoon. In the deep part of the carbonate aquifer, the chemistry of groundwater is mostly influenced by the intrusion of Tyrrhenian seawater, while in the superficial part, the input of saline lagoon water is predominant.

The Hg concentrations in groundwater of the study area varied widely, often exceeding the contamination threshold (1 μg/L) established by Italian Legislative Decree 152/2006. Mercury levels in groundwater were not correlated with the percentage of saline water, depth in the aquifer, or distance from the lagoon. These findings exclude Tyrrhenian seawater and saline water from the Lagoon of Orbetello as direct sources of Hg in local groundwater and responsible for release and mobilisation of the element by interaction with the carbonate lithologies hosting the aquifer.

The available data suggests that the Hg in groundwater of the Ansedonia coastal plain could come from surface geomaterials such as the Quaternary continental sediments that overlay the carbonate lithologies of the aquifer. This hypothesis is supported by the fact that i) high Hg concentrations were found in these coastal continental deposits as well as in contiguous lagoon sediments; ii) the highest Hg concentrations were found in groundwater from the upper part of carbonate aquifer; iii) Hg levels in groundwater increased with increasing thickness of the continental deposits. Mercury in the continental and lagoon sediments can be considered geogenic and attributed to i) regional and local sources of Hg related to geochemical anomalies in southern Tuscany (e.g. Monte Amiata mercury district); ii) sedimentary and pedogenetic processes responsible for the distribution and accumulation of Hg-rich particles in the continental sediments as well as formation of Hg-enriched eluvial deposits and residual soils.

It therefore seems likely that i) the Quaternary continental sediments and contiguous lagoon sediments are the main source of Hg in groundwater of the Ansedonia coastal plain; ii) waters circulating in these sediments dissolve the Hg-bearing solid components (e.g. cinnabar, Fe–Mn oxyhydroxides and organic matter) and mobilise the element presumably as chloride complexes; iii) the Hg-enriched waters move from the continental and lagoon sediments into the upper part of the carbonate aquifer, drawn by the cone of depression generated by intense pumping of groundwater from wells by the fish farms in the Ansedonia area.

To define the main factors and processes responsible for Hg release from the solid constituents of Quaternary continental and lagoon sediments and for its solubility and mobility in groundwater, further studies on Hg fractionation in these sediments and Hg speciation in groundwater and lagoon water are needed.

## Supplementary Information

Below is the link to the electronic supplementary material.

Supplementary file1 (PDF 704 KB)

## Data Availability

The datasets used and/or analysed during the current study are available on reasonable request.
