# Supplementary material for: New geochemical data for defining origin and distribution of mercury in groundwater of a coastal area in southern Tuscany (Italy)
Source: Environ Sci Pollut Res Int. 2023 Feb 18;30(17):50920–37. doi: 10.1007/s11356-023-25897-7 (PMC10104938; doi:10.1007/s11356-023-25897-7)
Supplement: Supplementary file 1 — Supplementary file1 (PDF 704 KB) [file 11356_2023_25897_MOESM1_ESM.pdf]

Supplementary Information

New geochemical data for defining origin and distribution of mercury in groundwater of a coastal area in southern Tuscany (Italy)

Giuseppe Protano[1*], Stefano Bianchi[2], Matteo De Santis[1], Luigi Antonello Di Lella[1], Francesco Nannoni[1], Massimo Salleolini[1]

[1] Department of Physical, Earth and Environmental Sciences, University of Siena, Via del Laterino 8, 53100 Siena, Italy
[2] Geologist, Via Roma 99, 58022 Follonica, Italy

* Corresponding author:
Protano Giuseppe - email: giuseppe.protano@unisi.it

Figures

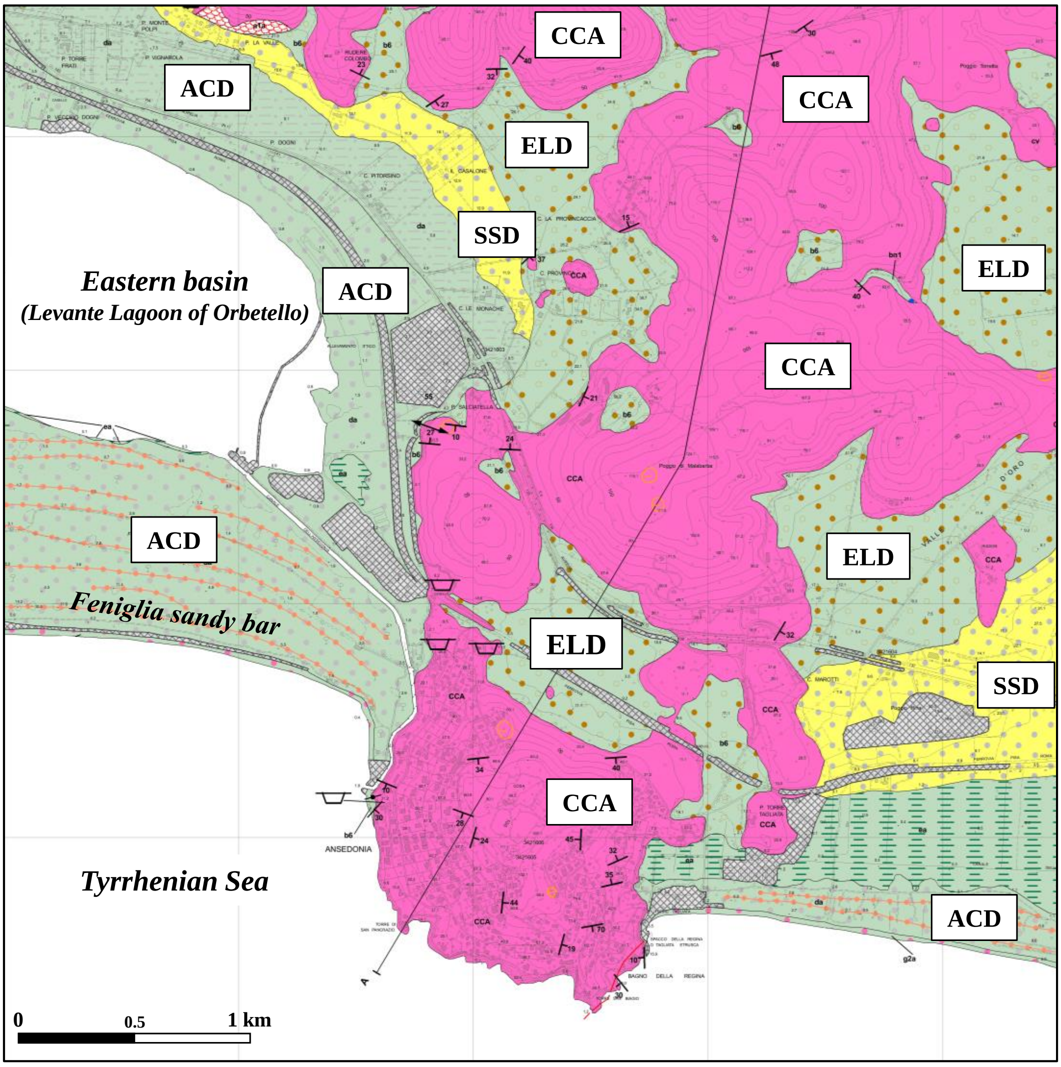

Fig. S1 Geological map of the study area (modified by Geological Map of Tuscany - Section 342160-Orbetello, scale 1:10000)

ELD: eluvial deposits (Holocene continental deposits)

ACD: aeolian coastal dunes (Holocene continental deposits)

SSD: stratified sandy dunes (Middle-Upper Pleistocene continental deposits)

CCA: Calcare cavernoso formation (Tuscan Nappe; Upper Triassic)

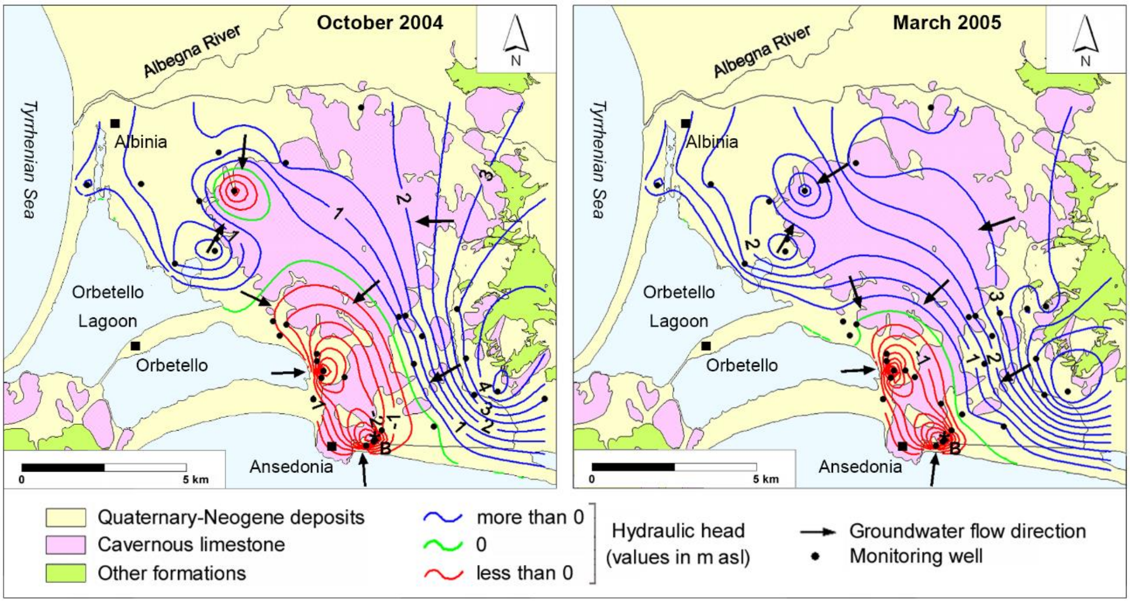

Fig. S2 Groundwater level contour maps for the carbonate aquifer system (redrawn from Nocchi and Salleolini, 2009)

Nocchi M, Salleolini M (2009) Simulating the impact of coastal pumping for fish farming purposes on the quality of groundwater: a case study in southern Tuscany, Italy. Proc. 2nd International FEFLOW User Conference, Potsdam/Berlin

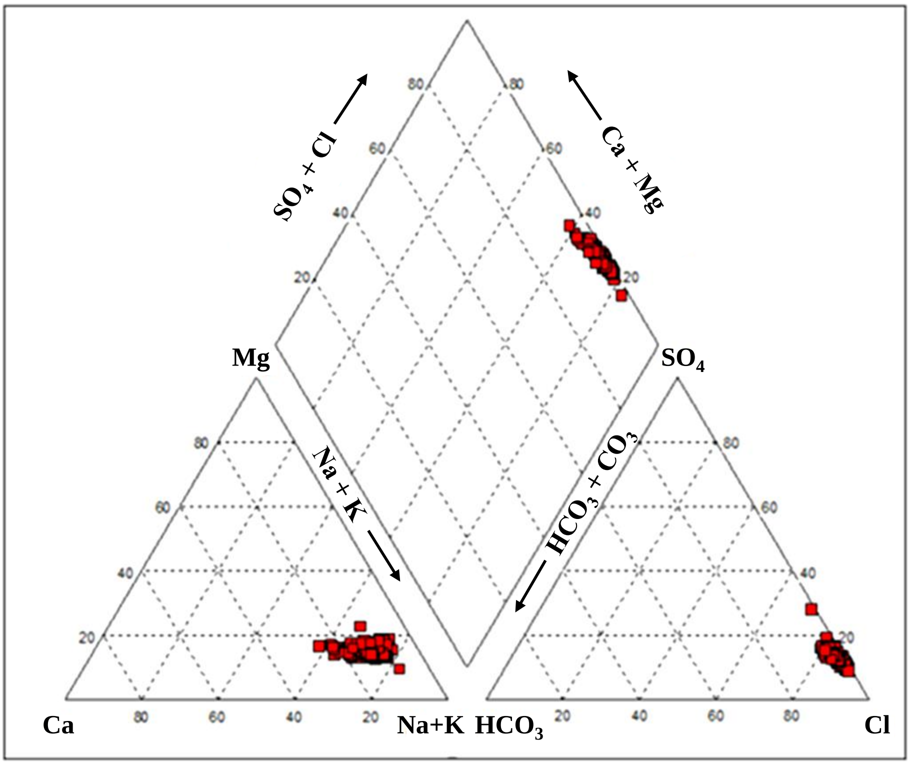

Fig. S3 Piper diagram showing the relationship between the major ion concentrations (average values) in groundwater of the study area

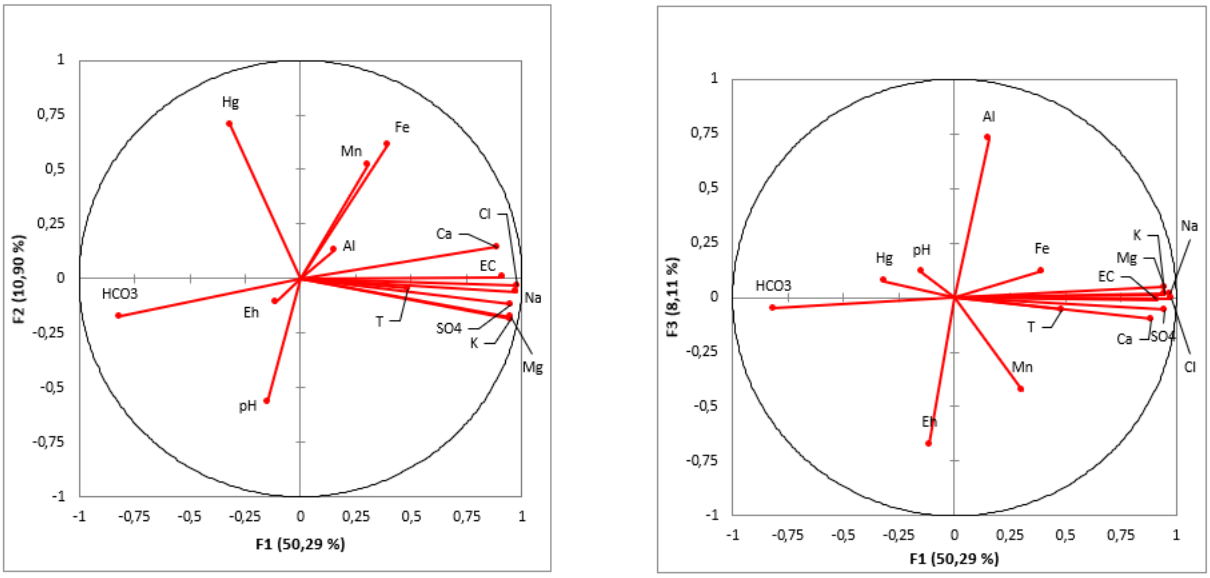

Fig. S4 Principal component analysis (PCA) biplots showing the loadings (vectors) of the physico-chemical properties and major and trace element concentrations in groundwater of the study area.

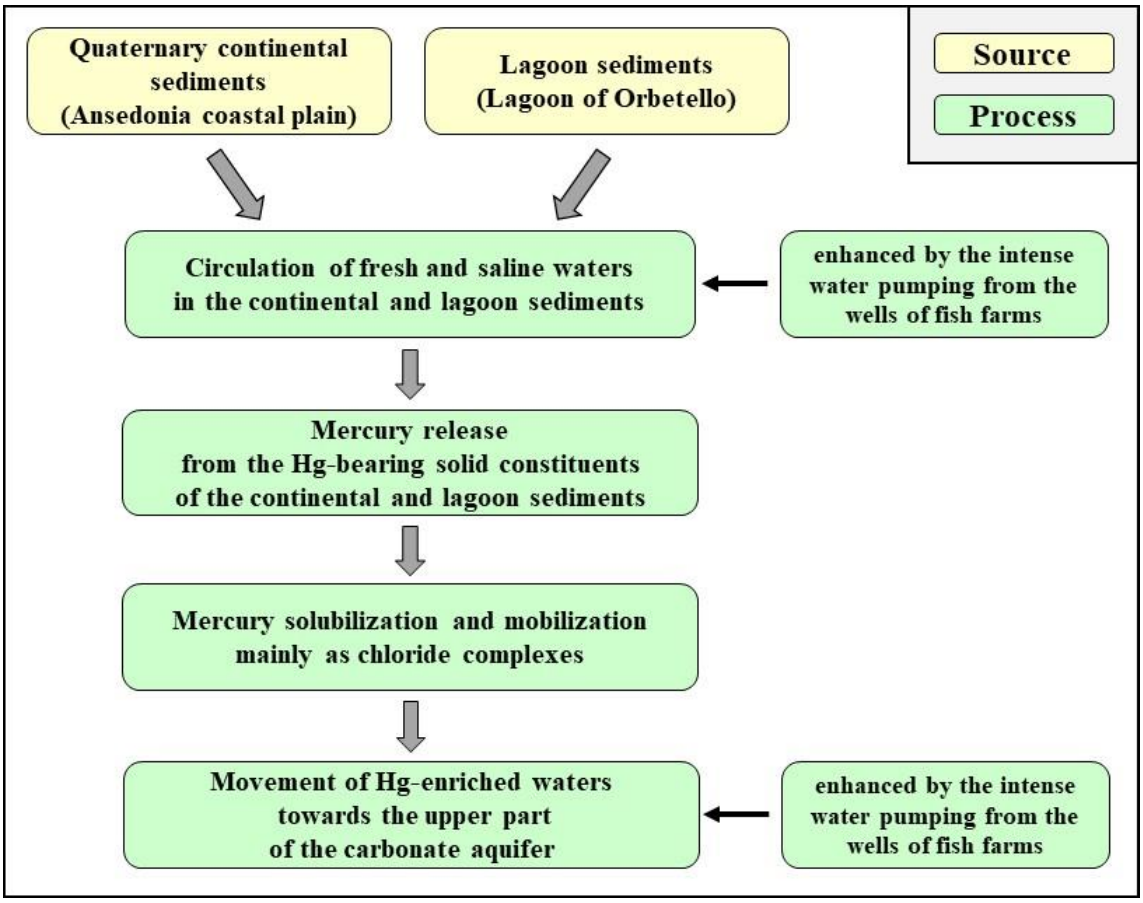

Fig. S5 Conceptual diagram reporting the sources and processes responsible for the Hg enrichment in groundwater of the Ansedonia coastal plain in the Orbetello Lagoon area.

Tables

Table S1 Statistical indices of pH, electrical conductivity, and major ion concentrations in groundwater of Groups A, B, C and D (N=number of data; EC=electrical conductivity at 20 °C; SD=standard deviation)

**Group A**	**pH**	**EC** *(μS/cm)*	**Na** *(mg/L)*	**K** *(mg/L)*	**Ca** *(mg/L)*	**Mg** *(mg/L)*	**Cl** *(mg/L)*	**HCO_3** *(mg/L)*	**SO_4** *(mg/L)*
**N**	79	76	77	73	77	78	78	79	77
**min**	6.5	8440	1313	38.9	289	186	2842	234	657
**max**	7.3	29500	6440	408	1137	638	10825	336	2430
**mean**	7.0	17979	3271	112	622	391	6233	284	1304
**SD**	0.2	4489	1026	54.7	157	108	1789	20.2	339
**25th percentile**	6.8	15560	2646	75.5	530	317	5190	272	1061
**50th percentile**	7.0	17610	3077	107	590	382	5910	284	1276
**75th percentile**	7.1	20403	3796	136	667	469	7558	297	1496

**Group B**	**pH**	**EC** *(μS/cm)*	**Na** *(mg/L)*	**K** *(mg/L)*	**Ca** *(mg/L)*	**Mg** *(mg/L)*	**Cl** *(mg/L)*	**HCO_3** *(mg/L)*	**SO_4** *(mg/L)*
**N**	60	61	61	59	61	61	61	61	59
**min**	6.5	16300	3276	116	443	407	7104	232	1003
**max**	7.3	42200	9712	342	1298	956	18633	323	3101
**mean**	7.0	27777	5551	191	759	643	10504	273	1933
**SD**	0.2	5942	1147	49.8	182	131	2025	21.2	382
**25th percentile**	6.9	24500	4812	160	631	531	9201	256	1759
**50th percentile**	7.0	27800	5355	175	734	637	10285	271	1881
**75th percentile**	7.1	31900	6171	221	859	762	11524	287	2047

**Group C**	**pH**	**EC** *(μS/cm)*	**Na** *(mg/L)*	**K** *(mg/L)*	**Ca** *(mg/L)*	**Mg** *(mg/L)*	**Cl** *(mg/L)*	**HCO_3** *(mg/L)*	**SO_4** *(mg/L)*
**N**	20	22	22	22	22	22	22	22	22
**min**	6.5	22000	4033	110	508	438	7394	217	1566
**max**	7.1	53500	10027	412	1467	1217	18217	293	4522
**mean**	6.9	39186	7692	263	1074	838	14867	245	2646
**SD**	0.2	8107	1254	69.9	229	171	2389	19.2	621
**25th percentile**	6.8	35973	7079	222	944	728	13954	232	2279
**50th percentile**	7.0	39350	7925	263	1070	832	14695	244	2490
**75th percentile**	7.0	43889	8314	284	1219	950	16592	256	2917

**Group D**	**pH**	**EC** *(μS/cm)*	**Na** *(mg/L)*	**K** *(mg/L)*	**Ca** *(mg/L)*	**Mg** *(mg/L)*	**Cl** *(mg/L)*	**HCO_3** *(mg/L)*	**SO_4** *(mg/L)*
**N**	15	14	15	14	14	14	15	15	15
**min**	6.6	34500	8470	339	1194	1006	14830	171	3126
**max**	7.2	62111	11636	559	1774	1259	22666	244	4601
**mean**	7.0	52153	10549	391	1351	1115	19584	193	3697
**SD**	0.2	7993	881	56.3	157	88.4	1899	17.6	389
**25th percentile**	6.9	51250	10297	356	1238	1032	18761	183	3453
**50th percentile**	6.9	52315	10774	378	1331	1110	19395	190	3565
**75th percentile**	7.1	57956	10922	396	1409	1177	20992	201	3971

Table S2 Values of physico-chemical properties (pH and electrical conductivity) and concentrations of major ions (Na, K, Ca, Mg, Cl, HCO_3, SO_4,) and trace elements (Hg, Fe, Mn, As, Sb) in lagoon water samples collected in the eastern part of the eastern basin of the Lagoon of Orbetello (EC=electrical conductivity at 20 °C)

Sample	pH	EC *(μS/cm)*	Na *(mg/L)*	K *(mg/L)*	Ca *(mg/L)*	Mg *(mg/L)*	Cl *(mg/L)*	HCO_3 *(mg/L)*	SO_4 *(mg/L)*
**L1**	8.57	52800	11659	351	556	1235	20149	214	2486
**L2**	8.30	50900	11210	341	568	1211	19525	204	2454
**L3**	8.19	48500	10761	332	599	1131	17966	223	2290
**L4**	7.96	42600	8962	273	651	936	15770	253	2171
**L5**	8.09	47100	10262	303	636	1067	17361	238	2267
**L6**	7.39	33400	7180	200	762	698	11745	272	1826
**L7**	7.94	43400	9270	281	653	962	16130	250	2181
**L8**	7.83	40200	8777	263	693	891	15185	262	2021

Sample	Hg *(μg/L)*	Fe *(μg/L)*	Mn *(μg/L)*	As *(μg/L)*	Sb *(μg/L)*
**L1**	<0.10	10.05	16.20	0.57	0.98
**L2**	<0.10	13.61	22.93	1.34	1.03
**L3**	<0.10	16.11	28.55	1.49	0.82
**L4**	<0.10	16.27	36.44	0.72	0.94
**L5**	0.16	16.49	28.41	1.38	0.88
**L6**	0.49	22.75	14.67	0.20	0.61
**L7**	<0.10	16.18	34.11	0.11	0.82
**L8**	0.15	13.32	35.14	0.30	0.83

Table S3 Concentrations of Hg, As and Sb in stream sediment samples collected along watercourses flowing in the Ansedonia coastal plain

Sample	Hg *(μg/L)*	As *(μg/L)*	Sb *(μg/L)*
**S1**	3.80	24.77	32.14
**S2**	1.90	28.98	15.89
**S3**	2.39	32.19	18.32
**S4**	1.00	44.85	12.01
**S5**	0.97	28.31	10.75
**S6**	3.36	29.55	14.02
